# Development of 8–17 XNAzymes that are functional in cells†

**DOI:** 10.1039/d3sc01928d

**Published:** 2023-06-28

**Authors:** Kosuke Chiba, Takao Yamaguchi, Satoshi Obika

**Affiliations:** a Graduate School of Pharmaceutical Sciences, Osaka University 1-6 Yamadaoka Suita Osaka 565-0871 Japan yamaguchi-ta@phs.osaka-u.ac.jp obika@phs.osaka-u.ac.jp; b National Institutes of Biomedical Innovation, Health and Nutrition 7-6-8 Saito-Asagi Ibaraki Osaka 567-0085 Japan; c Institute for Open and Transdisciplinary Research Initiatives (OTRI), Osaka University 1-1 Yamadaoka Suita Osaka 565-0871 Japan

## Abstract

DNA enzymes (DNAzymes), which cleave target RNA with high specificity, have been widely investigated as potential oligonucleotide-based therapeutics. Recently, xeno-nucleic acid (XNA)-modified DNAzymes (XNAzymes), exhibiting cleavage activity in cultured cells, have been developed. However, a versatile approach to modify XNAzymes that function in cells has not yet been established. Here, we report an X-ray crystal structure-based approach to modify 8–17 DNAzymes; this approach enables us to effectively locate suitable XNAs to modify. Our approach, combined with a modification strategy used in designing antisense oligonucleotides, rationally designed 8–17 XNAzyme (“X8–17”) that achieved high potency in terms of RNA cleavage and biostability against nucleases. X8–17, modified with 2′-*O*-methyl RNA, locked nucleic acid and phosphorothioate, successfully induced endogenous *MALAT-1* and *SRB1* RNA knockdown in cells. This approach may help in developing XNAzyme-based novel therapeutic agents.

## Abstract 2

Modified 8–17 DNAzymes that can induce target gene silencing in cells were developed based on the X-ray crystal structure of 8–17 DNAzyme and the modifications used in designing antisense oligonucleotides.

## Graphical abstract

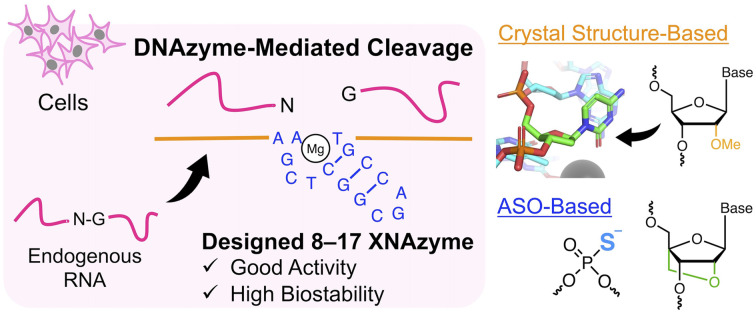

## Introduction

DNA enzymes (DNAzymes), first discovered in 1994,^1^ have attracted much attention as specific metal ion sensors,^2,3^ oligonucleotide ligation catalysts,^4,5^ and therapeutic modalities that cleave substrate RNA.^6,7^ These single-strand DNAs, often 30 nucleotides (nt) long, perform various functions, including hybridizing the complementary RNA strand, fixing metal ions as a catalyst, and causing various reactions against target RNAs (like RNA cleavage or ligation).^8–10^ Hence, DNAzymes are considered unique nucleic acids and are actively researched.

Antisense oligonucleotides (ASOs) and siRNA have been developed as RNA-cleaving nucleic acids and some have been approved as nucleic acid-based drugs.^11,12^ DNAzymes are unique in that, unlike the recruitment of RNase H or argonautes by ASOs and siRNA, they do not need to recruit endogenous enzymes. DNAzymes bind to target RNA in a similar manner as ASOs; *via* metal-ion catalysis, DNAzymes catalyze the intra-molecular nucleophilic attack on the RNA phosphodiester bond by RNA 2′-hydroxy group, coordinated by their catalytic core.^8^ This unique characteristic could be applied to the treatment of infectious diseases and cancer,^13–16^ which modify cellular conditions and endogenous enzyme activity. Although clinical research on the application of DNAzymes against basal-cell carcinoma or asthma has been conducted,^17,18^ there is limited evidence that DNAzymes mediate catalytic RNA cleavage in cells. Moreover, like ASOs, DNAzymes have been shown to cleave target RNA *via* RNase H.^19^

Recently, some studies have reported xeno-nucleic acid (XNA)-modified DNAzymes (XNAzymes) that exhibit activity in cells.^16,20–23^ Wang *et al.*^20^ reported a 10–23 XNAzyme exhibited RNA cleavage activity in cells. The negative controls, in which the sequence of the catalytic core was inverted to deactivate the DNAzyme, could not cleave the target RNA, demonstrating that DNAzyme activity was not an RNase H-mediated antisense effect. The authors modified DNAzyme with 2′-fluoroarabino nucleic acid (FANA) and α-l-threofuranosyl nucleic acid, finding that sufficient modification, especially of the binding arms, dramatically improves DNAzyme biostability and enhances RNA cleavage activity in cells.^20^ There has been extensive research on DNAzyme catalytic core modification.^20,24–27^ For example, Schubert *et al.*^26^ reported an approach in which each nucleotide at the catalytic core of 10–23 DNAzyme was replaced *via* 2′-*O*-methyl (2′-OMe) sequential modification, revealing that the site of the modification affects DNAzyme activity. It is important to modify the catalytic core to enhance biostability without reducing its activity. However, there remains considerable uncertainty regarding the effective design of the catalytic core.

Here, we describe a new approach to modifying the catalytic core based on its X-ray crystal structure. Structural biology provides powerful tools for revealing the dynamics of biological molecules. Recently, Borggräfe *et al.*^28^ clarified the catalytic process of 10–23 DNAzyme *via* real-time NMR measurements and successfully improved the cleavage activity of 10–23 DNAzyme by eliminating detrimental interaction between Mg^2+^ and guanine (G14) at the catalytic core, using 6-thioguanine modification.

In this study, we focus on the 8–17 DNAzyme, which, along with the 10–23 DNAzyme, was discovered *via in vitro* selection.^6^ The fine X-ray crystal structure of 8–17 DNAzyme, thought to reflect the precatalytic form, was reported in 2017.^29^ We presumed that the crystal structure would enable us to select appropriate nucleotides for modification with XNA, based on the characteristics of each nucleotide at the catalytic core. This approach does not require evaluation of all positions of the catalytic core, making it possible to effectively and rapidly generate high-potency DNAzymes at low cost. Furthermore, we tried to enhance the intracellular RNA-cleaving activity of 8–17 DNAzyme. To promote DNAzyme biostability against nuclease digestion, we used an ASO-based design approach to modify the binding arms of the 8–17 DNAzyme. The modified 8–17 DNAzyme (8–17 XNAzyme), hereafter denoted as “X8–17”, exhibited sufficient biostability and RNA-cleaving activity, even within cells.

## Results and discussion

### Optimizing the catalytic core based on the X-ray crystal structure

**Fig. 1:**
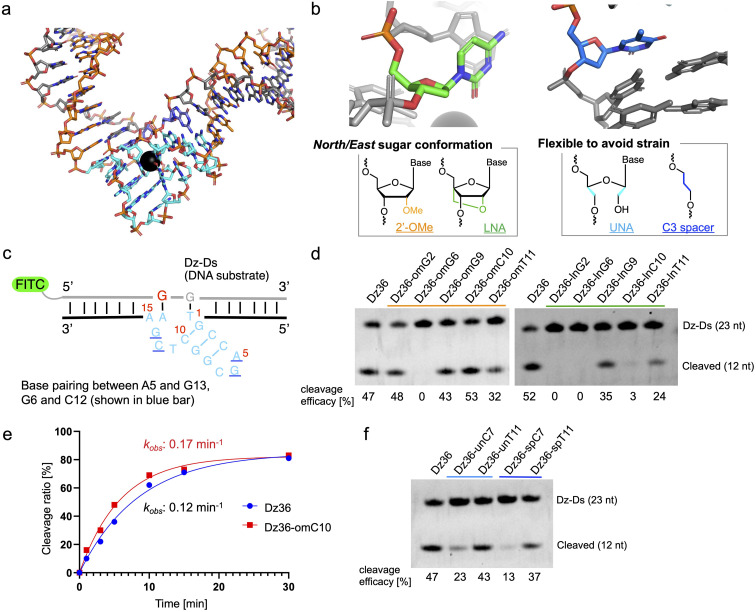
Chemical modification of the catalytic core of 8–17 DNAzyme based on X-ray crystal structure. (a) X-ray crystal structure of the 8–17 DNAzyme (unmodified sequence: Dz36). Orange: binding arms; blue: catalytic core; grey: DNA substrate. The black ball in the catalytic core is Pb^2+^. (b) Crystal structure-based chemical modification; *North*/*East* conformation nucleoside (left, C10 shown in light green) and strained nucleoside without base-pairing (right, T11 shown in blue). (c) Scheme of the cleavage assay of the X8–17. The DNA substrate was modified with FITC at the 5′ end, and only its cleavage site comprised RNA (guanine, shown in red). Urea-PAGE analysis revealed the substrate-cleavage activity under multiple-turnover conditions. The results are shown for (d) *North*/*East* conformation-induced modification and (f) modification to induce nucleotide flexibility. The cleavage reactions were performed in buffer containing 0.25 mM Pb(OAc)_2_ at 37 °C with 100 nM of the substrate and 10 nM of DNAzymes. (e) Time-dependent cleavage analysis of Dz36 and Dz36-omC10. The observed pseudo first-order rate constant (*k*_obs_) is shown. Dz-Ds: DNA substrate for Dz36 sequence.

The X-ray crystal structure of 8–17 DNAzyme, reported by a previous study, reflects the probable precatalytic form at high resolution (Fig. 1a, Protein Data Bank accession 5XM8).^29^ Based on the crystal structure, 8–17 DNAzyme hybridizes the DNA substrate *via* two binding arms (Fig. 1a, orange) and forms a catalytic core (15 nt) between the two arms (Fig. 1a, light blue). The catalytic core is a complex higher-order structure formed by base-pairing. Within the complex catalytic core of 8–17 DNAzyme, we focused on (i) nucleotides with a *North*/*East* sugar conformation (pseudorotation phase angle: 0°–108°) and (ii) nucleotides which do not perform base-pairing (Fig. 1b). It was expected that 2′-OMe RNA and locked nucleic acid (LNA), which induce a *North* sugar conformation, would be suitable for nucleotides with a *North*/*East* sugar conformation. For the modification of nucleotides which do not perform base-pairing, unlocked nucleic acid (UNA) and C3 spacer were selected to make the nucleotides flexible by eliminating the strain of sugar conformation. Such modifications have been reported to increase biostability against nucleases.^30–33^

To evaluate the effectiveness of this crystal structure-based chemical modification, a 5′-fluorescein isothiocyanate (FITC) modified DNA substrate in which the cleavage site was substituted with RNA (hereafter denoted as “Dz-Ds”) was used for the cleavage reaction (Fig. 1c). The substrate sequence that we used was identical to that of the X-ray crystal structure of 5XM8, with a DNA complementary strand. We refer to the unmodified 8–17 DNAzyme sequence (36 nt) as Dz36. First, we performed a cleavage assay under multiple turnover conditions (Dz36 : Dz-Ds = 1 : 10) where Dz36 could cleave *ca.* 50% of the substrate (under incubation at 37 °C for 10 min in physiological buffer containing 0.25 mM Pb(OAc)_2_; Pb(OAc)_2_ was used because the X-ray crystal structure was obtained using Pb^2+^ as the metal ion catalyst).

The results of the cleavage assay are shown in Fig. 1d. As expected, by substituting the nucleotides with a *North*/*East* sugar conformation *via* 2′-OMe modification, DNAzyme activity was retained in most cases, except for Dz36-omG6, which was modified with 2′-OMe at the G6 position in the catalytic core (Fig. 1d, lane 3). The G6 position, the sixth nucleotide from the 5′ end in the catalytic core, is highly essential for stabilizing Pb^2+^, so even a small conformational change could be critical at this position.^29^ Nonetheless, four out of the five selected nucleotides with the 2′-OMe modification were well-tolerated. In addition, the cleavage efficacy was slightly improved (by *ca.* 13%) at the C10 position (Dz36-omC10), relative to that of Dz36. We further compared the time-dependency of the cleavage rates of Dz36 and Dz36-omC10 (Fig. 1f) and also evaluated Michaelis–Menten kinetics (ESI Fig. 1†). The Dz36-omC10 rate constant (*k*_obs_) was slightly higher, and the Michaelis–Menten constant (*K*_m_) was smaller than that of Dz36, revealing its relatively better affinity to Dz-Ds and higher substrate cleavage activity. In contrast, LNA modifications were not tolerated, and almost all the X8–17s exhibited diminished activity, except for Dz36-lnG9, modified with LNA at G9 position (Fig. 1d, right). To clarify this further, we calculated the sugar puckering of each nucleotide at the catalytic core, based on the X-ray crystal structure (ESI Table 2†).^29^ The selected nucleotides exhibited the *North*/*East* sugar conformation (pseudorotation phase angle: 0°–108°). LNA modification induced the *North* conformation more than 2′-OMe modification, by fixing the C2′–C4′ bond (ESI Table 2, lower panel†). This abnormally fixed each nucleotide with the *North*/*East* sugar conformation and thereby potentially impaired DNAzyme activity. It is also plausible that the LNA structure is conformationally unfavorable for interaction with Pb^2+^.

For UNA and C3 spacer modification of nucleotides that do not perform base-pairing, modified at T11 position retained its DNAzyme activity after modification (Fig. 1e, Dz36-unT11 [UNA] and Dz36-spT11 [C3 spacer]), indicating that the T11 position did not need a sugar structure and nucleobase. However, in a previous study, the complete deletion of T11 reduced DNAzyme activity by approximately 20-fold.^29^ Our results indicate the usefulness of replacing non-base-pairing nucleotides with a UNA or C3 spacer to increase biostability.

Moreover, to improve Dz36 biostability, we synthesized X8–17s with two points of modification in the catalytic core (ESI Fig. 2†); the X8–17s modified in this way retained their DNAzyme activity. However, for further analysis, we selected Dz36-omC10, which exhibited relatively higher DNAzyme activity (Fig. 1d and e).

### Optimizing substrate-binding arms by following the general ASO wing design

To improve Dz36 potency, we focused on its affinity for its substrate. For this purpose, we modified the binding arms of Dz36. We hypothesized that the role of the binding arms is similar to the role in ASOs, which bind to complementary RNA strands with suitable turnover and are stable *in vivo*. Therefore, chemical modifications reported in ASO design could be useful for DNAzymes.

**Fig. 2:**
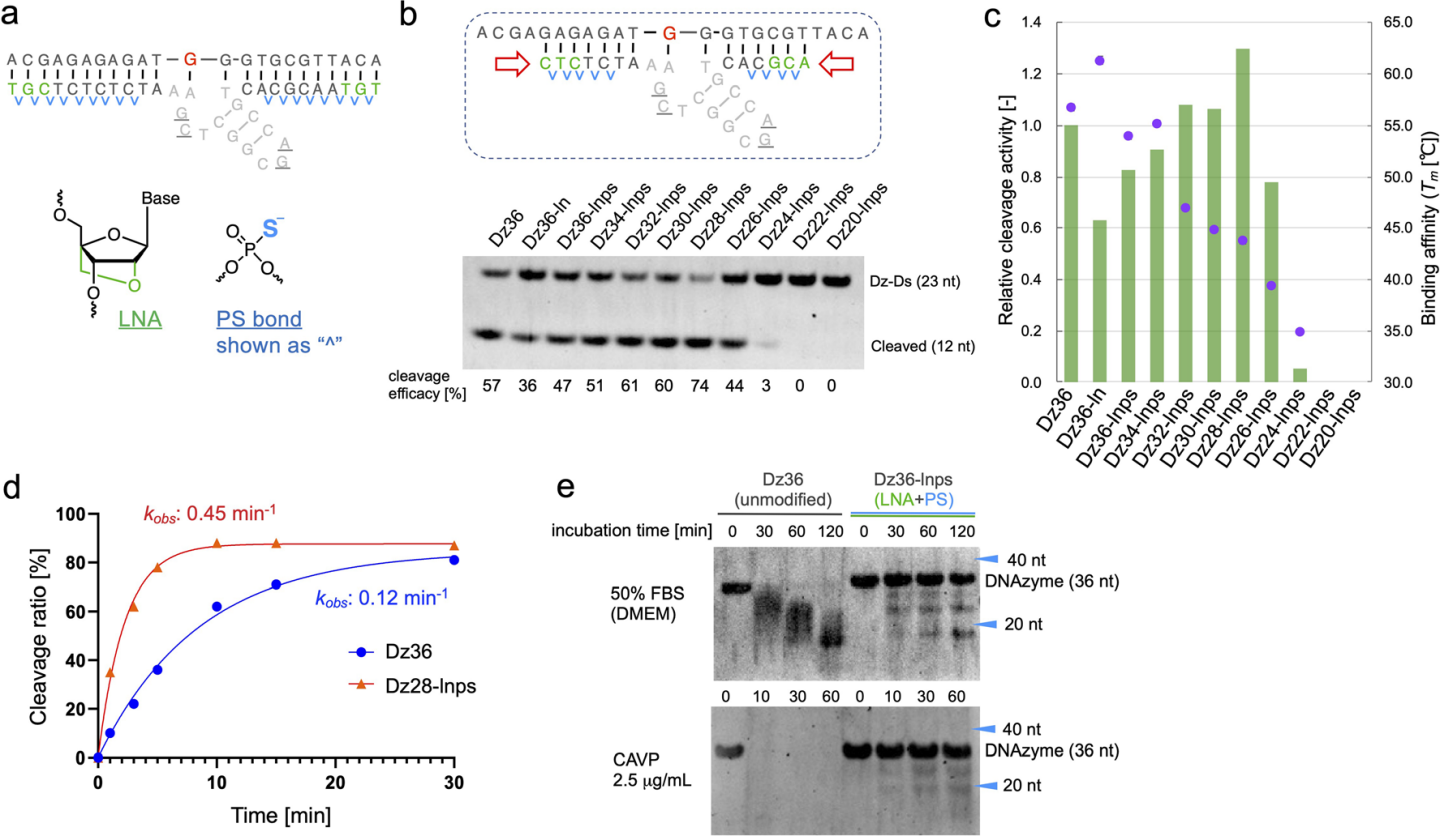
Optimizing the binding arms of Dz36, using modifications generally used for ASO design. (a) ASO-based design of the binding arms using LNA and PS modifications for binding affinity and biostability. (b) Activity of the X8–17s with modified binding arms. The ASO-based design and the length of the binding arms were tested under the standard cleavage condition. (c) Relationship between the cleavage activity and binding affinity (*T*_m_ value). Cleavage activity (bar) was calculated relative to that of Dz36 (based on the results in panel (b)). Dots: *T*_m_ values. (d) Time-dependent cleavage analysis of Dz36 and Dz28-lnps. (e) Biostability assay to compare Dz36 and Dz36-lnps (modified based on ASO design) in 50% FBS (top) or 2.5 μg mL^−1^*Crotalus adamanteus* venom phosphodiesterase (CAVP) (bottom).

LNA is frequently applied to the 5′ and 3′ ends of ASOs, called the wing regions. LNA improves exonuclease resistance and binding affinity against the complementary strand.^33^ The phosphorothioate (PS) bond, a good alternative to the naturally occurring phosphodiester (PO) bond, is used in almost all linkages of ASO to improve its biostability against DNA nucleases.^34^ Similarly, Taylor *et al.*^22^ incorporated several PS bonds in the FR6_1 DNAzyme. Based on these, we applied LNA and PS bonds to the binding arms of Dz36 (Fig. 2a). As expected, the LNA and PS modified Dz36, hereafter referred to as “Dz36-lnps”, worked well and its activity was retained (Fig. 2b, lane 3), whereas LNA modification without PS (Dz36-ln) exhibited reduced activity relative to Dz36. Moreover, we presumed that adequate cleavage activity requires an optimal binding arm length, based on the cleavage mechanism (proper binding affinity and turnover against substrates). To test this hypothesis, arm length was investigated by shortening them one by one from both ends with Dz36-lnps (Fig. 2b, right). The shortened 28 nt derivative (“Dz28-lnps”) exhibited the highest cleavage activity (57% higher than that of Dz36-lnps).

To establish the connection between binding affinity and cleavage efficacy, the thermal stability, denoted by melting temperature (*T*_m_) values, of X8–17s and the substrate were measured (Fig. 2c). The *T*_m_ values and corresponding cleavage activity levels were inversely correlated from Dz36-lnps to Dz28-lnps. These findings reveal that shortening the binding arms reduced affinity toward the substrate, resulting in efficient cleavage activity due to improved turnover. When the binding arm was shorter than 26 nt (“Dz26-lnps”), *T*_m_ was below 35 °C and cleavage activity was almost absent. It was hypothesized that Dz24-lnps, Dz22-lnps, and Dz20-lnps could not even bind to the substrate under normal physiological conditions (cleavage assay temperature: 37 °C). These results are congruent with those of a previous report suggesting that *T*_m_ should ideally be close to the working temperature.^26^ Moreover, the *T*_m_ for Dz36-ln was 5 °C higher than that for Dz36, which was also reflected in the cleavage activity, with Dz36-ln exhibiting lower activity than Dz36 and Dz36-lnps, owing to its insufficient turnover arising from its higher thermal stability. This could be explained by the fact that LNA modifications raise binding affinity, whereas PS modifications slightly reduce this affinity.^35,36^ Next, we performed kinetic studies to compare the unmodified Dz36 and Dz28-lnps, which exhibited the highest cleavage activity (Fig. 2d; ESI Fig. 1†). Notably, Dz28-lnps exhibited almost 4-fold higher *k*_obs_ value than Dz36 in multiple-turnover assays. In addition, it was revealed that Dz28-lnps had >2-fold higher *V*_0_ and *k*_cat_ than Dz36. These results indicate that turnover was substantially improved by adjusting binding affinity toward the substrate and that our strategy to shorten the binding arms was reasonable.

The biostability of Dz36-lnps, modified with LNA and PS, was assessed by comparing it with that of unmodified Dz36 (Fig. 2e). In the presence of 50% fetal bovine serum (FBS) or phosphodiesterase, Dz36-lnps exhibited much higher biostability than Dz36, as expected, indicating that these modifications worked well in terms of biostability. We further evaluated the stability of Dz28-lnps along with that of Dz36 and Dz36-lnps (ESI Fig. 3†) and found that Dz28-lnps also exhibited good biostability.

### Confirming the effect of metal ions and the difference between DNA and RNA substrates

Based on our findings, we expected X8–17 with both modified catalytic cores and binding arms to be functional in living cells. Prior to evaluating RNA knockdown activity in cells, it was important to confirm whether our design could be applied to an RNA substrate and in the presence of Mg^2+^ or Zn^2+^, which reflect intracellular conditions.

**Fig. 3:**
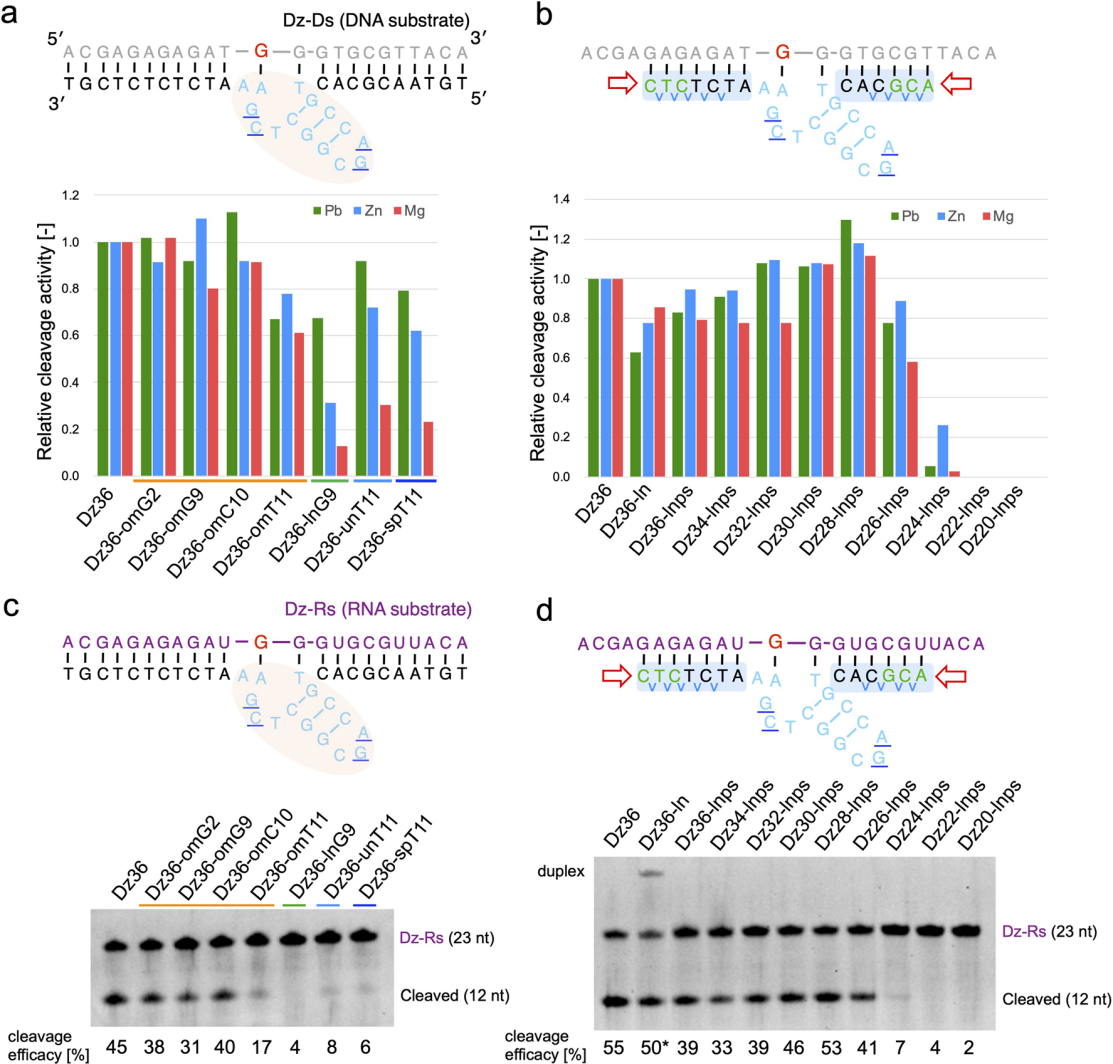
Effects of metal ions on Dz36 modification. Metal ion dependency is illustrated for (a) catalytic core modifications and (b) binding arm modifications. Substrate-cleavage reactions were performed under standard condition with either 0.25 mM Pb(OAc)_2_, 2.5 mM ZnCl_2_, or 2.5 mM MgCl_2_. Cleavage activity was normalized by setting the activity of Dz36 to 1.0. The results for Pb^2+^ were analyzed using the data in Fig. 1d, f and 2b. The cleavage assay used an RNA substrate in the presence of 2.5 mM MgCl_2_. Modifications of (c) the catalytic core and (d) the binding arms were applied to the RNA substrate. Dz36-ln cleavage activity was used as the reference value (represented by *) because of its insufficient dissociation of the duplex. Dz-Rs: RNA substrate.

We first tested the effect of using different metal ions, whereby Pb^2+^ was replaced with Mg^2+^ or Zn^2+^ (Fig. 3a and b). When modifying the catalytic core, the difference in metal ion strongly affected X8–17 cleavage activity (Fig. 3a). The 2′-OMe-modified X8–17s exhibited good tolerance of the change in metal ion, whereas the other X8–17s exhibited reduced activity, especially under Mg^2+^ conditions. The differences in the impact of each modification might be due to differences in the catalytic core structure. 8–17 DNAzyme is known to folds into a more compact structure in the presence of Mg^2+^ or Zn^2+^ than in the presence of Pb^2+^.^37,38^ Therefore, X-ray crystal structure-based modification of DNAzymes might be limited the effectiveness when the structure is slightly altered by replacing with Mg^2+^ or Zn^2+^.

In contrast, binding arm modification was well tolerated in the Mg^2+^ condition (Fig. 3b). The trends in cleavage activity were almost the same, and Dz28-lnps (at 28 nt, with a shorter binding arm) exhibited the best activity. From these results, it can be inferred that changing the metal ions strongly affected catalytic core modifications but did not affect binding arm modifications.

We next evaluated the effects of PS modification. “Dz36-ps_full” (*i.e.*, Dz36 with an entirely PS modifications), exhibited significantly reduced activity under Zn^2+^ or Mg^2+^ conditions (ESI Fig. 4b†). PS bonds are known to be more hydrophobic than PO bonds. In addition, the catalytic core phosphate bonds all face outward (toward the aqueous media).^29^ We speculated that the catalytic core with PS bonds becomes unstable in aqueous media, especially with additional folding induced by Zn^2+^ or Mg^2+^. It is also possible that PS modification destabilized the electrostatic interaction between a coordinated metal ion and the 5′-PO bond of A5 (distance: 5.1 Å).^29^ In contrast, Dz36-ps, with PS modifications only in its binding arm, exhibited almost the same activity as Dz36 even under Mg^2+^ conditions (ESI Fig. 4b†). Based on these results, we applied PS modifications only to the binding arms and not to the catalytic core (Fig. 2a). Further investigations are warranted concerning PS modifications in the catalytic core.

To demonstrate X8–17 activity in cells, the RNA substrate, not the DNA substrate, should be cleaved. Hence, we performed the same experiments using the RNA substrate (Dz-Rs) in the presence of Mg^2+^ (Fig. 3c and d). The 2′-OMe modification of the catalytic core and binding arm length optimization were similarly effective for the RNA substrate. Introducing chemical modifications without reducing RNA-cleavage activity is expected to improve biostability and the possibility of intracellular RNA cleavage. Moreover, *T*_m_ values were measured; the cleavage activity and binding affinity were correlated for the Dz-Rs as for the Dz-Ds (ESI Fig. 5†).

### Evaluation of endogenous RNA cleavage activity in cells

**Fig. 4:**
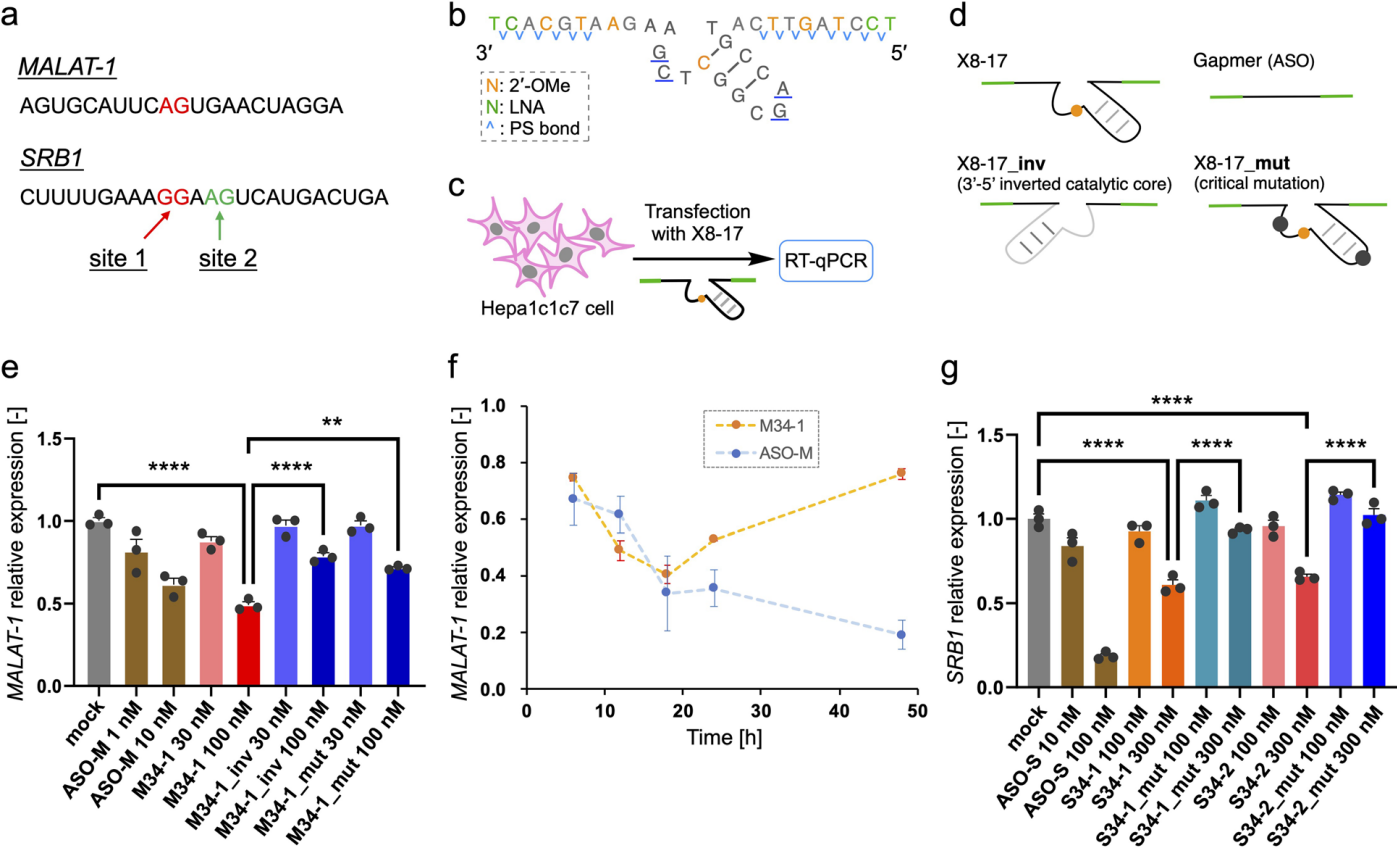
Assay of endogenous RNA knockdown by X8–17s. (a) Reported ASO-targeting sequence of *MALAT-1* and *SRB1* RNA. The cleavage site of X8–17s is shown in red and green. (b) The design of M34-1, which targets *MALAT-1*; S34-1 and S34-2, targeting *SRB1*, had the same design. (c) Scheme of an endogenous RNA knockdown assay. (d) Images of X8–17 and their negative controls “inv” and “mut”, and of the ASO gapmer. (e) Endogenous *MALAT-1* knockdown assay, 12 h after transfection, using one-way ANOVA and Dunnett's test (**P < 0.01, ****P < 0.0001). (f) Time-dependent knockdown activity of M34-1 from 6 to 48 h post-transfection. The knockdown assay results of ASO-M (10 nM) and M34-1 (100 nM) were shown. (g) Endogenous *SRB1* knockdown assay at 12 h post-transfection, *via* one-way ANOVA and Tukey's test (****P < 0.0001). (e–g) Error bars: mean ± standard deviation for *n* = 3 independent samples.

Finally, we investigated the target RNA cleavage activity of X8–17 inside cultured cells. A non-coding RNA metastasis-associated lung adenocarcinoma transcript 1 (*MALAT-1*), which is widely used for ASO studies,^39^ was selected as the model endogenous RNA. Dz36 preferentially cleaves a A–G dinucleotide to the same extent as a G–G dinucleotide;^40^ therefore, we designed X8–17 by targeting the A–G dinucleotide, which is located near the ASO binding site (Fig. 4a, shown in red).

For the X8–17 targeting *MALAT-1*, we used 2′-OMe modification at C10 of the catalytic core, and applied LNA and 2′-OMe modifications to the binding arms incorporated with PS bonds (M34-1, Fig. 2c). It is possible that an unmodified region or only the PS-modified region would be recognized by RNase H, with its target RNA cleaved *via* the antisense effect. RNase H is known to recognize continuous DNA/RNA sequences.^41,42^ Therefore, 2′-OMe modifications were applied as alternate nucleotides to avoid RNase H recognition. In terms of its binding affinity, LNA modification increases *T*_m_ by *ca.* 4–6 °C per modification,^35^ whereas 2′-OMe modification increases it by *ca.* 0.5–1.5 °C.^43^ Hence, we changed the design described in Fig. 2a to include three 2′-OMe modifications instead of one LNA modification in the binding arm (Fig. 4b). We confirmed that the cleavage activity of these designs was comparable (M34-1, M34-2; ESI Fig. 6b†). Furthermore, the binding arm length of *MALAT-1* targeting M34-1 and M34-2 (34 nt) was designed based on the calculated *T*_m_ value, which was close to that for Dz28-lnps, and was selected based on cleavage activity (ESI Fig. 6b†).

In the negative control (M34-1_inv), the sequence of the catalytic core was inverted, modifying the type of negative control used by Wang *et al.*^20^ However, Taylor *et al.*^44^ and Spitale *et al.*^45^ have revealed that a negative control inactivated *via* point mutations in the catalytic core is better than inversion of the catalytic core sequence because it causes minimal perturbation. Hence, we used another negative control sequence (M34-1_mut) inactivated by a two-point mutation of the catalytic core, which reduces Dz36 activity (Fig. 4d).^29^ Gel-electrophoresis analysis confirmed that neither negative control cleaved its substrate (ESI Fig. 6b†). In addition, M34-1 successfully exhibited sequence-specific cleavage of A–G dinucleotides against a 70 nt substrate of *MALAT-1* partial RNA (M-Rs-2, ESI Fig. 6c†).

Intracellular knockdown assays were performed using Hepa1c1c7 cells. M34-1s and previously reported *MALAT-1* targeting ASO (ASO-M) were transfected, followed by knockdown analysis using quantitative reverse transcription (qRT)-PCR (Fig. 4c). *GAPDH* was used as a housekeeping gene and *MALAT-1* expression was corrected using the ΔΔ*C*_t_ method. M34-1 exhibited dose-responsive *MALAT-1* knockdown with significant differences relative to the negative controls (Fig. 4e). These results indicate that the knockdown activity of M34-1 was DNAzyme-mediated. Meanwhile, we found that even the negative controls, M34-1_inv and M34-1_mut, exhibited some knockdown activity at 100 nM, implying the occurrence of RNase H-mediated knockdown. Therefore, the knockdown activity of M34-1 may involve the antisense effect of RNase H, along with DNAzyme-mediated knockdown.

Moreover, we evaluated the activity of M34-2, which had longer unmodified DNA sequences at the binding arms (ESI Fig. 7a and b†). Although M34-2 exhibited knockdown activity, this was almost the same as that of the corresponding negative control M34-2_inv. These results suggest that X8–17 has a potential antisense effect owing to its unmodified binding arms. In contrast, M34-1, with reduced RNase H recognition sites in the binding arms *via* LNA and 2′-OMe modifications, exhibited successful DNAzyme-mediated knockdown.

M34-1 activity became weaker with an increase in incubation time (Fig. 4f). This reflects the instability of X8–17 in biological fluids. Dz36-lnps, modified with LNA and PS bonds, exhibited significantly improved biostability relative to Dz36 (Fig. 2e). After 6 h in FBS only 30% of the Dz36-lnps remained, while after 24 h of incubation almost all of the Dz36-lnps had disappeared (ESI Fig. 8a and b†). This instability is due to the fact that the PS bonds were not applied to the catalytic core and that X8–17 is easily degraded by endonucleases. Further study is needed to obtain long-lasting X8–17-mediated knockdown activity in cells.

Next, we tried to establish the versatility of X8–17-mediated knockdown activity in cells. The scavenger receptor B1 (*SRB1*), which is also known as ASO-targeting RNA,^46^ was chosen as a coding RNA. We designed a 34 nt long X8–17 at the G–G and A–G dinucleotide sites around the ASO-targeting sequence (S34-1 and S34-2, Fig. 4a). We then confirmed the site-specific cleavage of them and the inactivity of the negative controls using gel-electrophoresis (ESI Fig. 9b and c†). The intracellular knockdown assay was performed in almost the same manner as in the case of *MALAT-1*; as the reference, *SRB1* targeting ASO (ASO-S) (previously reported^46^) was assessed (Fig. 4g). Both S34-1 and S34-2 exhibited dose-dependent *SRB1* knockdown activity. Remarkably, the negative controls with two-point mutation of the catalytic core exhibited negligible activity, indicating clearly that S34-1 and S34-2 exhibited DNAzyme-mediated knockdown without the antisense effect.

As demonstrated, X8–17s successfully exhibited DNAzyme-mediated knockdown activity for two kinds of endogenous RNA, *MALAT-1* and *SRB1*, in cultured cells. The knockdown activity by X8–17s differed significantly from that of the negative controls that were prepared to distinguish DNAzyme-mediated cleavage from RNase H-mediated cleavage. Further, we experimentally confirmed that the degree of RNase H-mediated cleavage was almost the same for X8–17s and the negative controls (ESI Fig. 10†). In case of the negative controls for *SRB1*, S34-1_mut and S34-2_mut did not exhibit any intracellular knockdown activity. These results were inconsistent with those shown in ESI Fig. 10,† which reveals RNase H-mediated cleavage with S34-1_mut and S34-2_mut. This is probably because the human RNase H1 concentration (10 ng μL^−1^) used in the assays is greater than the intracellular concentration (0.5–2.5 ng μL^−1^),^23^ and the enzyme is possibly more reactive in buffer conditions than in crowded cellular conditions. To the best of our knowledge, this is the first study clearly demonstrating intracellular cleavage activity of 8–17 DNAzyme.

In summary, based on the X-ray crystal structure of the 8–17 DNAzyme, we effectively modified its catalytic core without reducing its activity. Further, ASO design enabled us to improve DNAzyme biostability and cleavage activity. The chemically modified X8–17s demonstrated endogenous RNA-knockdown activity in cultured cells. These findings therefore highlight the effectiveness of chemical modification at suitable sites in 8–17 DNAzyme. This approach can also be applied to other nucleic acid enzymes.

DNAzyme has great potential to effectively mediate allele-selective RNA knockdown.^21,22^ It is known that 8–17 DNAzyme preferentially cleaves the N–G dinucleotide (where “N” indicates all four nucleobases in the following order of preference: G > A > C > U).^40^ In contrast, the 10–23 DNAzyme preferentially cleaves G–U,^20^ and FR6_1, developed by Taylor *et al.*,^22^ cleaves almost all dinucleotides that have adenosine one nucleotide downstream. The X8–17 designed here thus broadens range of dinucleotides that can be cleaved intracellularly by DNAzyme.

Several potential limitations need to be considered. First, the RNA-knockdown activity of our X8–17s was *ca.* 10 times weaker than that of ASOs evaluated at the same time. The cleavage activity of M34-1, S34-1, and S34-2 was lower than that of the original Dz36 (Fig. 3c; ESI Fig. 6 and 9†). Consequently, optimization of each X8–17 sequence was necessary to approach the knockdown activity of ASOs. Second, X8–17 exhibited instability within 24 h post-transfection (Fig. 4f; ESI Fig. 11†). Therefore, to improve its biostability against nucleases, there is a need for catalytic core modifications, such as PS modifications, *South* conformation-induced modifications such as FANA, and *North* type modifications. Here, we focused on the *North*-type sugar conformations seen in 8–17 DNAzyme, as we have been researching ASO chemistry in which 2′-OMe, MOE, and LNA are commonly used. We suggest that highly modified DNAzymes developed in future could have great potential *in vitro* and even *in vivo*, thereby promoting future therapeutic modalities.

## Methods

### Materials

The oligonucleotide sequences used in the study are shown in ESI Table 1.† The DNA and RNA oligonucleotides (including DNA primers and complementary strands) were synthesized and purified by GeneDesign Inc. (Osaka, Japan) or Hokkaido System Science Co., Ltd (Sapporo, Japan).

### DNAzyme activity *in vitro*

Each DNAzyme was first annealed to its 5′-FITC labeled complementary strand (substrate) for 3 min at 90 °C in 100 mM Hepes buffer (pH 7.2) and gradually cooled to 26 °C for 2 h. DNAzyme cleavage assay was performed in 100 mM Hepes buffer (pH 7.3) containing 400 mM KCl, 100 mM NaCl, and 0.25 mM Pb(OAc)_2_ (or 2.5 mM ZnCl_2_, or 2.5 mM MgCl_2_) at 37 °C. The cleavage reaction was quenched by adding loading buffer (90% formamide, 40 mM EDTA, 0.01% xylene cyanol) at each time-point and was heated for 3 min at 95 °C. The samples were analyzed using 4 M urea and 15% PAGE in 0.5× TBE buffer. The FITC-labeled substrates were visualized using the iBright FL1500 imaging system (Thermo Fisher Scientific, Waltham, MA) and quantified using ImageJ 1.53 (National Institutes of Health, Bethesda, MD). For the DNA substrate (in which the cleavage site was substituted with an RNA substrate), the DNAzyme concentration was 10 nM and 25 nM in the case of the RNA substrate. In each case, 100 nM of substrate was used. *k*_obs_ was calculated by fitting the amount of cleaved substrate as the percentage of the total substrate and the reaction time (min), using the Prism 9.5.0 (GraphPad Software, San Diego, CA) (eqn (1)):

1 *R*_*t*_ = *R*_∞_(1 − e^−*k*_obs_*t*^)

where *R*_*t*_ is the percentage of the cleaved substrate at time *t* and *R*_∞_ is the apparent reaction plateau.

### Sugar puckering assessment

Sugar puckering was calculated based on the reported X-ray crystal structure^27^ using Pymol 2.4.0 (Schrödinger, New York, NY). Pseudorotation phase angle (*P*_p_) and *ν*_max_ were calculated using eqn (2) and (3), respectively:2
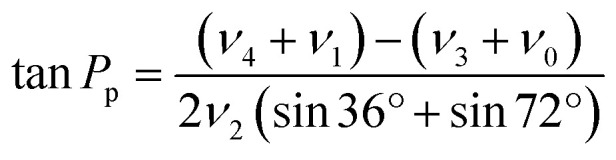

3 *ν*_max_ = *ν*_2_/cos *P*_p_

where *ν*_0_, *ν*_1_, *ν*_2_, *ν*_3_, and *ν*_4_ are the endocyclic torsion angles of the five-membered ring (sugar structure of the nucleotide) defined as follows:*ν*_0_: C4′–O4′–C1′–C2′*ν*_1_: O4′–C1′–C2′–C3′*ν*_2_: C1′–C2′–C3′–C4′*ν*_3_: C2′–C3′–C4′–O4′*ν*_4_: C3′–C4′–O4′–C1′

### *T*_m_ analysis

*T*_m_ was measured using the UV-1650PC and UV-1800 spectrometers (Shimadzu, Kyoto, Japan) equipped with a *T*_m_ analysis accessory. Each DNAzyme and its complementary DNA (acgagagagatgggtgcgttaca) or RNA (ACGAGAGAGAUgGGUGCGUUACA) strand (lower case: DNA, upper case: RNA) was dissolved in 100 mM Hepes buffer (pH 7.3) containing 400 mM KCl and 100 mM NaCl to a final concentration of 2 μM. Absorbance was measured at 260 nm from 20 to 95 °C at a scan rate of 0.5 °C min^−1^. *T*_m_ was obtained from the melting curve as the temperature of the half-dissociation of the formed duplexes.

### Biostability assay

Each DNAzyme (1 μM) was incubated in 50% FBS and Dulbecco's modified Eagle medium (DMEM) solution at 37 °C and was collected at each time-point (0.5, 1, 2, 6, and 24 h) by quenching with stop and loading buffers (90% formamide, 40 mM EDTA, and 0.01% xylene cyanol), followed by denaturing for 5 min at 95 °C. The samples were analyzed using 15% denaturing PAGE with urea, and the gels were stained using SYBR Gold (Thermo Fisher Scientific), as per the manufacturer′s instructions. The stained gels were visualized using the iBright FL1500 system. For the nuclease stability assay, each DNAzyme (1 μM) was dissolved in 50 mM Tris–HCl buffer (pH 8.0) containing 10 mM MgCl_2_ and 2.5 μg mL^−1^*Crotalus adamanteus* venom phosphodiesterase at 37 °C; the same procedure was followed as for the 50% FBS experiment.

### Cell culture and X8–17 transfection

Hepa1c1c7 cells (ATCC, Manassas, VA) were cultured in low-glucose DMEM (WAKO Chemicals, Osaka, Japan) containing 10% FBS and 1× antibiotic–antimycotic solution for cell culture and were maintained in a 5% CO_2_ incubator at 37 °C. For X8–17 transfection, the cells were seeded 1 day before transfection, at a density of 5000 to 12 000 cells per well in Iwaki 96-well plates (AGC Techno Glass, Shizuoka, Japan), depending on the incubation time. After 24 h, the cells were transfected with X8–17s or ASOs, using Lipofectamine 3000, as per the manufacturer's instructions, and were further grown in high-glucose DMEM containing 10% FBS and 1× antibiotic–antimycotic solution. After transfection and incubation, the cells were harvested and used for assays.

### Quantification of *MALAT-1* RNA

After X8–17 transfection for each time-period, total RNA was isolated from the samples using the SuperPrep II Cell Lysis Kit (Toyobo, Osaka, Japan), according to the manufacturer's instructions. Next, total RNA was reverse transcribed using the SuperPrep II RT Kit (Toyobo). For qRT-PCR, the obtained cDNA and specific primer sets were used, along with the PowerTrack SYBR Green Master Mix (Thermo Fisher Scientific). qRT-PCR was performed using StepOnePlus (Applied Biosystems; Thermo Fisher Scientific), and the amplification specificity of the PCR products was assessed by analyzing the melting temperature curve of the qRT-PCR products. The forward and reverse primers for *MALAT-1* were 5′-ACATTCCTTGAGGTCGGCAA-3′ and 5′-CACCCGCAAAGGCCTACATA-3′, respectively. The housekeeping gene *GAPDH* was used as an internal control. The forward and reverse primers for *GAPDH* were 5′-TCACCACCATGGAGAAGGC-3′ and 5′-GCTAAGCAGTTGGTGGTGCA, respectively. The data were statistically analyzed using Prism 9.5.0 (GraphPad Software).

### Quantification of *SRB1* RNA

After X8–17 transfection for each time-period, *SRB1* RNA was assessed using the same procedure as for *MALAT-1* RNA estimation. Next, the obtained cDNA and specific TaqMan primer sets were mixed with the TaqMan Fast Advanced Master Mix (Applied Biosystems; Thermo Fisher Scientific), followed by qRT-PCR. The TaqMan probes for *GAPDH* (Mm99999915_g1) and *SRB1* (Mm00450234_m1) were purchased from Thermo Fisher Scientific. Data were statistically analyzed using Prism 9.5.0 (GraphPad Software).

### Initial velocity determination for Michaelis–Menten curve

The Michaelis–Menten curves for DNAzyme were determined by determining the initial velocity (*V*_0_ [nM min^−1^]). *V*_0_ was calculated from a linear fit of the DNAzyme-mediated cleavage reaction within the 10–25% cleavage range, using eqn (4):4
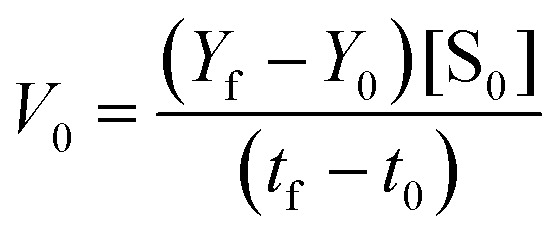
where *Y*_f_ is the percent cleavage at time *t*_f_ in the 10–25% cleavage range, *Y*_0_ is the percent cleavage at *t*_0_, and [S_0_] is the initial substrate concentration (in nM). Values of *V*_0_ were obtained at five concentrations of Dz-Ds (the DNA substrate) (25, 50, 250, 500, and 1000 nM) ([S]) with 10 nM DNAzyme ([E_0_]) in the presence of 0.25 mM Pb(OAc)_2_. *K*_m_ and *k*_cat_ were then calculated by fitting *V*_0_ and [S], using Prism 9.5.0 (GraphPad software) (eqn (5) and (6)):5
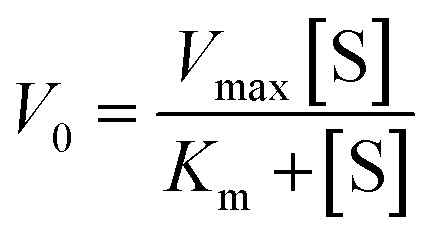
6
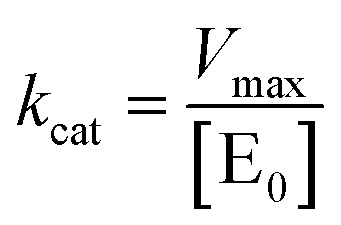


### RNase H1 assays

Co-annealed 0.13 μM X8–17s and 0.13 μM long substrate (70 nt) was incubated with 0.01 μg μL^−1^ human RNase H1 (Abcam, Cambridge, UK) in 100 mM Hepes buffer (pH 7.3) containing 400 mM KCl, 100 mM NaCl, and 1 mM MgCl_2_ (1 h at 37 °C). The reactions were quenched by adding an excess of loading buffer (90% formamide, 40 mM EDTA, 0.01% xylene cyanol) and heating for 5 min at 95 °C. The samples were analyzed by gel electrophoresis.

## Data availability

The datasets supporting this article have been uploaded as part of the ESI.†

## Author contributions

The experiments were designed by K. C., T. Y., and S. O., and were performed by K. C. The manuscript was written by K. C. and T. Y. All authors have approved the final version of the manuscript.

## Conflicts of interest

The authors declare no competing interests.

## Supplementary Material

SC-014-D3SC01928D-s001
