# Supplementary material for: Development of 8–17 XNAzymes that are functional in cells
Source: Chem Sci. 2023 Jun 28;14(28):7620–9. doi: 10.1039/d3sc01928d (PMC10355097; doi:10.1039/d3sc01928d)
Supplement: SC-014-D3SC01928D-s001 [file SC-014-D3SC01928D-s001.pdf]

Electronic Supplementary Material (ESI) for Chemical Science.
This journal is © The Royal Society of Chemistry 2023

Supplementary Information

Development of 8–17 XNAzymes that are functional in cells

Kosuke Chiba[1], Takao Yamaguchi[1,*], Satoshi Obika[1,2,3,*]

[1]*Graduate School of Pharmaceutical Sciences, Osaka University, 1-6 Yamadaoka, Suita, Osaka 565-0871, Japan*
[2]*National Institutes of Biomedical Innovation, Health and Nutrition, 7-6-8 Saito-Asagi, Ibaraki, Osaka 567-0085, Japan*
[3]*Institute for Open and Transdisciplinary Research Initiatives (OTRI), Osaka University, 1-1 Yamadaoka, Suita, Osaka 565-0871, Japan*

*Corresponding Authors: Takao Yamaguchi, yamaguchi-ta@phs.osaka-u.ac.jp; Satoshi Obika, obika@phs.osaka-u.ac.jp

Contents

1. Supplementary Tables page S2–S4
2. Supplementary Figures page S5–S16
3. Uncropped Full Gel Images page S17–S24
4. Supplementary References page S24

Supplementary Table 1. DNAzymes and substrates (oligonucleotides) used in this study.

	Oligonucleotide Name	Sequence (5′–3′)
	(Numbering)	tgtaacgcac–tgc-cag-cgg-ctc-gaa–atctctctcgt<br>1-3 4-6 7-9 10-12 13-15
Reported 8–17 DNAzyme X-ray crystal structure sequence[1] (**Dz**)		
	Dz36	tgtaacgcac–tgccagcggctcgaa–atctctctcgt
	Dz-Ds	(5′-FITC) acgagagagat**Gg**gtgcgttaca
	Dz-Rs	(5′-FITC) ACGAGAGAGAU**GG**GUGCGUUACA
	Dz-Ds- T_m	acgagagagatgggtgcgttaca
	Dz-Rs- T_m	ACGAGAGAGAUgGGUGCGUUACA
	Catalytic core Modification	
	Dz36-omG2	tgtaacgcac–tG^{M}c-cag-cgg-ctc-gaa–atctctctcgt
	Dz36-omG6	tgtaacgcac–tgc-caG^{M}-cgg-ctc-gaa–atctctctcgt
	Dz36-omG9	tgtaacgcac–tgc-cag-cgG^{M}-ctc-gaa–atctctctcgt
	Dz36-omC10	tgtaacgcac–tgc-cag-cgg-C^{M}tc-gaa–atctctctcgt
	Dz36-omT11	tgtaacgcac–tgc-cag-cgg-cT^{M}c-gaa–atctctctcgt
	Dz36-lnG2	tgtaacgcac–tG^{L}c-cag-cgg-ctc-gaa–atctctctcgt
	Dz36-lnG6	tgtaacgcac–tgc-caG^{L}-cgg-ctc-gaa–atctctctcgt
	Dz36-lnG9	tgtaacgcac–tgc-cag-cgG^{L}-ctc-gaa–atctctctcgt
	Dz36-lnC10	tgtaacgcac–tgc-cag-cgg-5^{L}tc-gaa–atctctctcgt
	Dz36-lnT11	tgtaacgcac–tgc-cag-cgg-cT^{L}c-gaa–atctctctcgt
	Dz36-unC7	tgtaacgcac–tgc-cag-C^{U}gg-ctc-gaa–atctctctcgt
	Dz36-unT11	tgtaacgcac–tgc-cag-cgg-cT^{U}c-gaa–atctctctcgt
	Dz36-spC7	tgtaacgcac–tgc-cag-(sp)gg-ctc-gaa–atctctctcgt
	Dz36-spT11	tgtaacgcac–tgc-cag-cgg-c(sp)c-gaa–atctctctcgt
	Dz36-flC10	tgtaacgcac–tgc-cag-cgg-C^{F}tc-gaa–atctctctcgt
	Dz36-moC10	tgtaacgcac–tgc-cag-cgg-5^{m}tc-gaa–atctctctcgt
	Dz36-omG9-omC10	tgtaacgcac–tgc-cag-cgG^{M}-C^{M}tc-gaa–atctctctcgt
	Dz36-flG9-omC10	tgtaacgcac–tgc-cag-cgG^{F}-C^{M}tc-gaa–atctctctcgt
	Dz36-omC10-unT11	tgtaacgcac–tgc-cag-cgg-$C^{M}T^{U}$c-gaa–atctctctcgt
	Dz36-omC10-spT11	tgtaacgcac–tgc-cag-cgg-C^{M}(sp)c-gaa–atctctctcgt
	Binding arm Modification	
	Dz36-ps_full	t^g^t^a^a^c^g^c^a^c^t^g^c^c^a^g^c^g^g^c^t^c^g^a^a^a^t^c^t^c^t^c^t^c^g^t
	Dz36-ps	t^g^t^a^a^c^g^c^ac–tgccagcggctcgaa–at^c^t^c^t^c^t^c^g^t
	Dz36-ln	$T^{L}G^{L}T^{L}$aacgcac–tgccagcggctcgaa–atctctct$5^{L}G^{L}T^{L}$

Dz36-lnps	T^{L}^G^{L}^T^{L}^a^a^c^g^c^ac–tgccagcggctcgaa–at^c^t^c^t^c^t^5^{L}^G^{L}^T^{L}
Dz34-lnps	G^{L}^T^{L}^A^{L}^a^c^g^c^ac–tgccagcggctcgaa–at^c^t^c^t^c^T^{L}^5^{L}^G^{L}
Dz32-lnps	T^{L}^A^{L}^A^{L}^c^g^c^ac–tgccagcggctcgaa–at^c^t^c^t^5^{L}^T^{L}^5^{L}
Dz30-lnps	A^{L}^A^{L}^5^{L}^g^c^ac–tgccagcggctcgaa–at^c^t^c^T^{L}^5^{L}^T^{L}
Dz28-lnps	A^{L}^5^{L}^G^{L}^c^ac–tgccagcggctcgaa–at^c^t^5^{L}^T^{L}^5^{L}
Dz26-lnps	5^{L}^G^{L}^5^{L}^ac–tgccagcggctcgaa–at^c^T^{L}^5^{L}^T^{L}
Dz24-lnps	G^{L}^5^{L}^A^{L}c–tgccagcggctcgaa–at^5^{L}^T^{L}^5^{L}
Dz22-lnps	5^{L}^A^{L}5^{L}–tgccagcggctcgaa–aT^{L}^5^{L}^T^{L}
Dz20-lnps	A^{L}5^{L}–T^{L}gccagcggctcgaa– A^{L}T^{L}^5^{L}
Dz36-om	T^{M}G^{M}T^{M}aacgcac–tgccagcggctcgaa–atctctctC^{M}G^{M}T^{M}
mouse MALAT-1 targeting DNAzyme (**M**)	
M34-1	T^{L}^5^{L}^c^T^{M}^a^G^{M}^t^T^{M}^catgccagcggC^{M}tcgaagA^{M}a^T^{M}^g^C^{M}^a^5^{L}^T^{L}
M34-1_inv	T^{L}^5^{L}^c^T^{M}^a^G^{M}^t^T^{M}^caaagctcggcgaccgtgA^{M}a^T^{M}^g^C^{M}^a^5^{L}^T^{L}
M34-1_mut	T^{L}^5^{L}^c^T^{M}^a^G^{M}^t^T^{M}^catgccaacggC^{M}ttgaagA^{M}a^T^{M}^g^C^{M}^a^5^{L}^T^{L}
M34-2	T^{L}^5^{L}^5^{L}^t^a^g^t^t^catgccagcggC^{M}tcgaagaa^t^g^c^A^{L}^5^{L}^T^{L}
M34-2_inv	T^{L}^5^{L}^5^{L}^t^a^g^t^t^caaagctcggcgaccgtgaa^t^g^c^A^{L}^5^{L}^T^{L}
M34-3	T^{L}^5^{L}^5^{L}^t^a^g^t^t^catgccagcggctcgaagaa^t^g^c^A^{L}^5^{L}^T^{L}
M32-3	5^{L}^5^{L}^T^{L}^a^g^t^t^catgccagcggctcgaagaa^t^g^5^{L}^A^{L}^5^{L}
M30-3	5^{L}^T^{L}^A^{L}^g^t^t^catgccagcggctcgaagaa^t^G^{L}^5^{L}^A^{L}
ASO-M	5^{L}^T^{L}^A^{L}^g^t^t^c^a^c^t^g^a^a^T^{L}^G^{L}^5^{L}
M-Rs-1	(5′-FITC) AGUGCAUUC**AG**UGAACUAGGA
M-Rs-2	(5′-FITC) GCAGCUGACCCAGGUCUACACAGAAGUGCAUUC**AG**UGAACUAG GAAGACAGGAGCGGCAGACAGGAGUCC
mouse SRB1 targeting DNAzyme (**S**)	
S34-1	G^{L}^T^{L}^c^A^{M}^t^G^{M}^a^C^{M}^tttgccagcggC^{M}tcgaatT^{M}t^C^{M}^a^A^{M}^a^A^{L}^G^{L}
S34-1_mut	G^{L}^T^{L}^c^A^{M}^t^G^{M}^a^C^{M}^tttgccaacggC^{M}ttgaatT^{M}t^C^{M}^a^A^{M}^a^A^{L}^G^{L}
S34-2	T^{L}^5^{L}^a^G^{M}^t^C^{M}^a^T^{M}^gatgccagcggC^{M}tcgaatC^{M}c^T^{M}^t^T^{M}^c^A^{L}^A^{L}
S34-2_mut	T^{L}^5^{L}^a^G^{M}^t^C^{M}^a^T^{M}^gatgccaacggC^{M}ttgaatC^{M}c^T^{M}^t^T^{M}^c^A^{L}^A^{L}
ASO-S	G^{m}^5^{m}^T^{m}^T^{m}^5^{m}^a^g^t^5^a^t^g^a^5^t^T^{m}^5^{m}^5^{m}^T^{m}^T^{m}
S-Rs-1	(5′-FITC) CTTTTGAAA**GG**A**AG**TCATGACTGA
S-Rs-2	(5′-FITC) ACUGGAAGAGAAAUUUUAUCAUCUUUUGAAA**GG**A**AG**UCAUGA CUGAAGCAAUAAACCUUUUCACUGAUUC

Lower cases letters: DNA residues; Upper case letters: RNA residues; N^{L}: LNA; N^{M}: 2′-OMe RNA; N^{U}: UNA; (sp): C3 spacer; N^{F}: 2′-F DNA; N^{m}: 2′-*O*-methoxyethyl (MOE) RNA; "5": 5-methyl cytidine; "^": phosphorothioate (PS) bonds. Blue font: DNA at the catalytic core of 8–17 DNAzyme. **Red font**: Critical mutation site for 8–17 DNAzyme. **Undelined**: cleavage site of substrates.

Supplementary Table 2. Calculation of sugar puckering for each nucleotide in the catalytic core of the 8–17 DNA enzyme (DNAzyme).

Calculations were conducted based on the X-ray crystal structure (Protein Data Bank [PDB] ID: 5XM83[1]). Five nucleotides (G2, G6, G9, C10, and T11) with the *North/East* type conformation were selected and were confirmed using the respective pseudorotation phase angle (P_p) and puckering amplitude (ν_{max}). Relative activity was calculated based on Fig. 1d, and the activity of the unmodified 8–17 DNAzyme (Dz36) was set as 1.0. For locked nucleic acid (LNA) and 2′-*O*-methyl (2′-OMe) sugar puckering calculations, one representative was selected from the X-ray structure in each case (PDB ID: 1HHW[2], an LNA–RNA hybrid; PDB ID: 1I7J[3], the dsRNA of 2′-OMe).

Position in Catalytic Core	P_p	υ_{max}	Prefered Conformation	Relative Activity 2′-OMe	Relative Activity LNA
G2	24.5	13.3	C3′ *endo*	**1.0**	< 0.1
G6	18.8	42.3	C3′ *endo*	< 0.1	< 0.1
G9	79.3	39.8	O4′ *endo*	**0.9**	**0.7**
C10	59.4	18.4	C4′ *exo*	**1.1**	< 0.1
T11	46.9	39.5	C4′ *exo*	**0.7**	< 0.1
LNA	16.3	57.5	C3′ *endo*	1HHW[2]	
2′-OMe	24.8	49.8	C3′ *endo*	1I7J[3]	

a

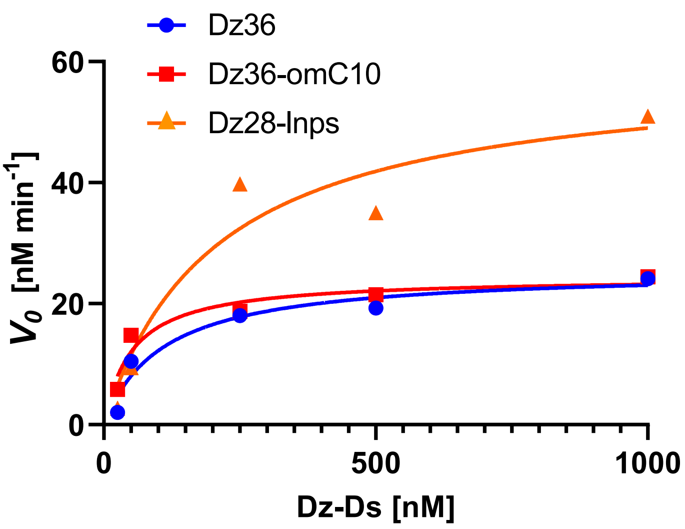

b

	K_m [nM]	k_{cat} [min^{-1}]	R square
Dz36	109.1	2.556	0.94
Dz36-omC10	51.42	2.438	0.92
Dz28-lnps	206.3	5.913	0.93

Supplementary Fig. 1. Michaelis-Menten curve of each DNAzyme.

a) Michaelis–Menten curves of Dz36, Dz36-omC10, and Dz28-lnps. Each curve reflects the initial velocity (V_0) plotted against the DNA substrate (Dz-Ds) concentration. b) The Michaelis–Menten constant (K_m) and k_{cat} of each DNAzyme were calculated from the plot in panel a.

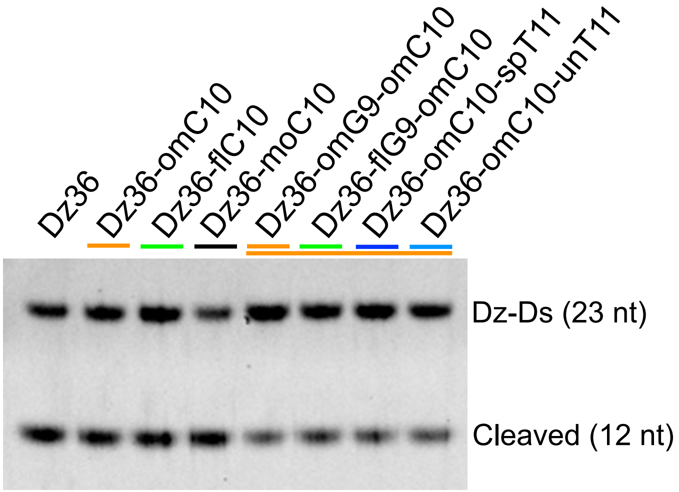

Supplementary Fig. 2. Differences between 2′-modifications and two-point modifications in the catalytic core.

The modifications at C10, using 2′-fluoro (Dz36-flC10) and 2′-*O*-methoxyethoxy (Dz36-moC10), had comparable effects on activity as those observed in Dz36-omC10. For the two-point modifications (lanes 5–8), while cleavage activity was retained, it was slightly less than that of the unmodified 8–17 DNAzyme (Dz36). Cleavage reaction conditions: 0.01 μM DNAzyme, 0.1 μM DNA substrate in 100 mM HEPES buffer (pH 7.3) with 0.25 mM $Pb(OAc)_2$, 400 mM KCl, and 100 mM of NaCl. The solution was incubated for 10 min at 37 °C.

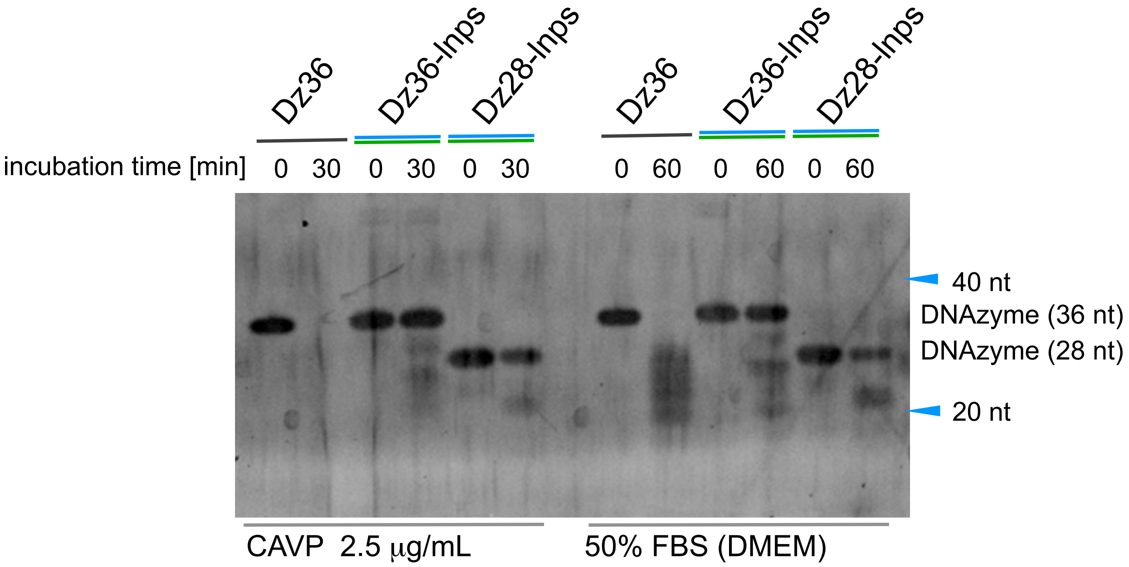

Supplementary Fig. 3. Biostability of Dz36, Dz36-lnps and Dz28-lnps.

An additional biostability assay was conducted to clarify the biostability of Dz28-lnps, as in the experiments shown in Fig. 2e. As expected, the 8–17 DNAzyme modified with LNA and PS modifications, Dz36-lnps and Dz28-lnps, exhibited substantially improved stability in 50% fetal bovine serum (FBS) or in the presence of phosphodiesterase (*Crotalus adamanteus venom phosphodiesterase* [CAVP]). In this experiment, the gels were stained with SYBR Gold.

a

	Modification	
	Catalytic core	Binding arm
Dz36	-	-
Dz36-ps_full	PS bond	PS bond
Dz36-ps	-	PS bond
Dz36-om	-	2′-OMe
Dz36-ln	-	LNA
Dz36-lnps	-	LNA / PS bond

b

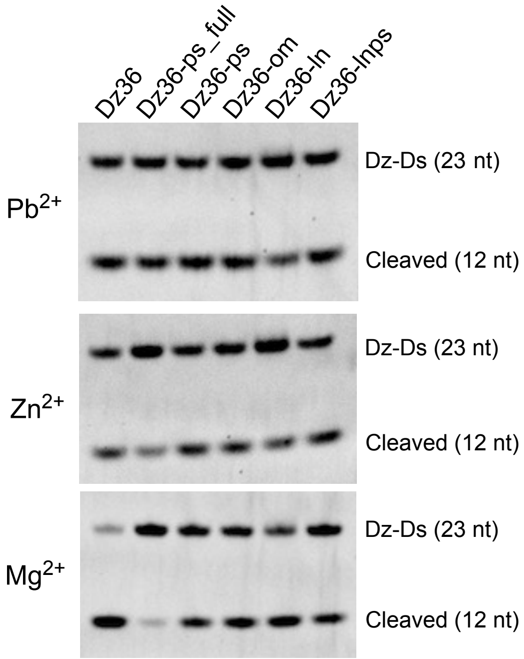

Supplementary Fig. 4. Effect of phosphorothioate (PS) and LNA/ 2′-OMe modifications of the binding arm of Dz36.

a) Modifications of assessed samples. The sequences are summarized in Supplementary Table 2. b) Cleavage activity in the presence of divalent metal ions (Pb^{2+}, Zn^{2+}, or Mg^{2+}). For Dz36-ps_full, the PS modifications for all nucleotides, including the catalytic core, exhibited reduced activity under Zn^{2+} and Mg^{2+} conditions; however, PS modifications at only the binding arm (Dz36-ps) was tolerated, as were other modified species, such as those with LNA and PS (Dz36-lnps). We chose LNA/PS as the binding arm modification, based on its expected high stability against nucleases. PS modification alone at the catalytic core was not used for further investigations. The cleavage assay was conducted under the same conditions as in Supplementary Fig. 1 but with different divalent ions concentrations and incubation times: (Pb^{2+}: 0.25 mM $Pb(OAc)_2$, 10 min; Zn^{2+}: 2.5 mM $ZnCl_2$, 20 min; Mg^{2+}: 2.5 mM $MgCl_2$, 60 min).

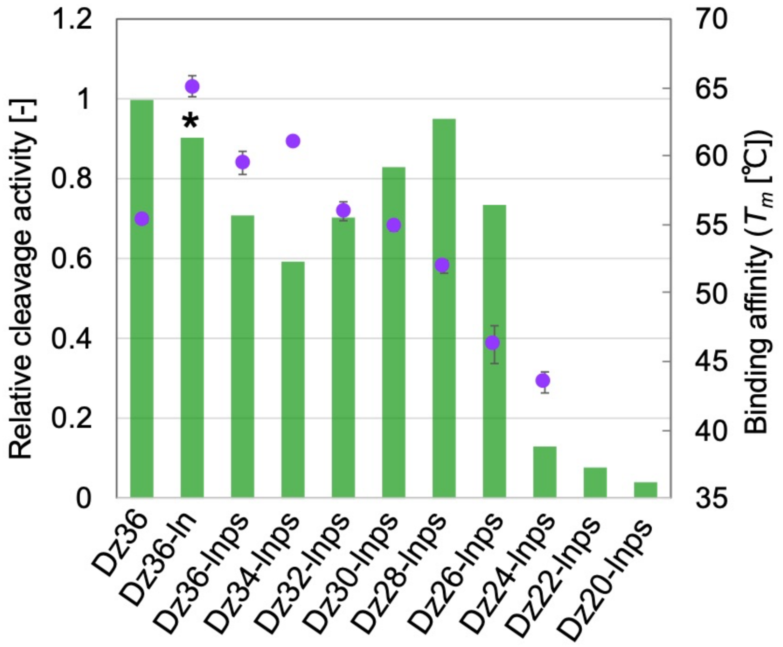

Supplementary Fig. 5. The association between cleavage activity and binding affinity for the RNA substrates.

The effect of modification and length on cleavage activity is shown for RNA substrates, analyzed using the data in Fig. 3d (bar). The dots indicate binding affinity (T_m). For the LNA modifications without PS (Dz36-ln), the cleavage activity represents the reference value, owing to insufficient duplex dissociation (represented by *). Cleavage reaction conditions: 0.025 µM DNAzyme, 0.1 µM RNA substrate in 100 mM HEPES buffer (pH 7.3) with 2.5 mM $MgCl_2$, 400 mM KCl, and 100 mM NaCl. The solution was incubated for 60 min at 37 °C.

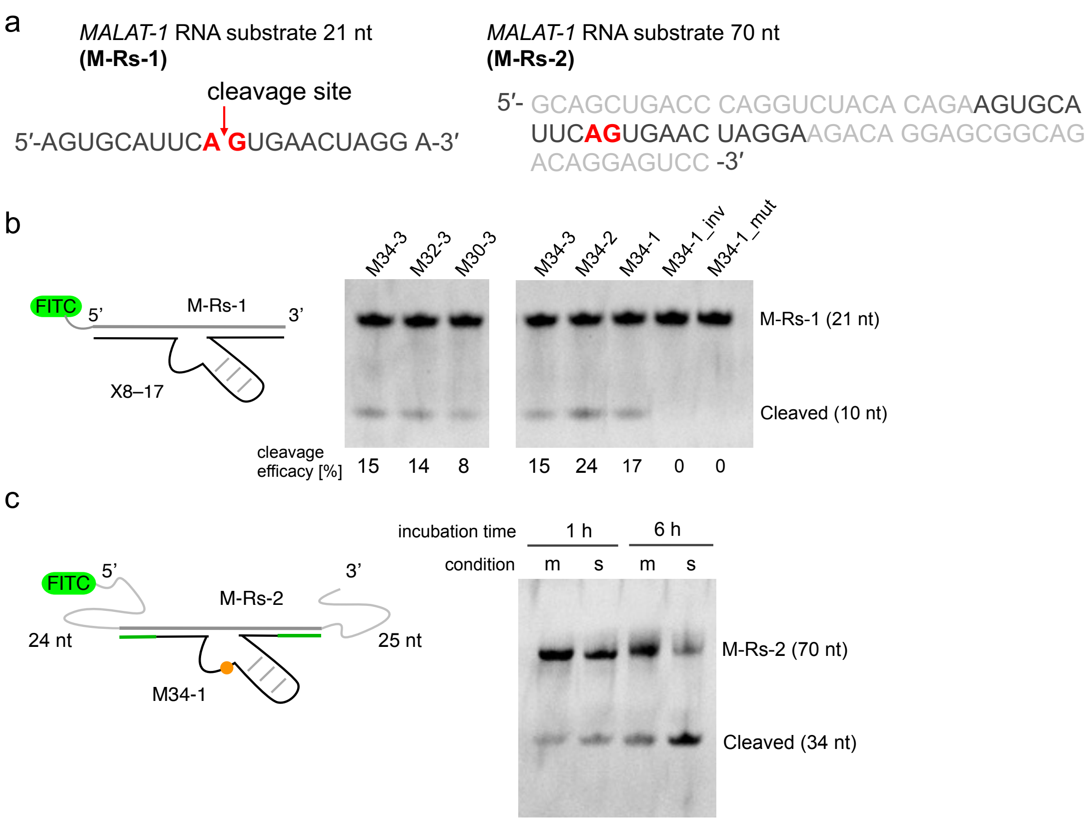

Supplementary Fig. 6. X8–17 mediated *MALAT-1* partial sequence cleavage.

a) *MALAT-1* partial sequence was used for the assay. Fluorescein isothiocyanate (FITC) was conjugated to its 5′ end. RNA substrates of 21 nt and 70 nt were prepared to confirm site-specific cleavage and the effects of the more structured substrate (secondary structure). The cleavage site is indicated in red. b) Cleavage assay. The binding arm was modified with LNA and PS bonds, and its length gradually reduced from both ends (M34-3, M32-3, and M30-3; sequences shown in Supplementary Table 2). The 34 nt length (M34-3) was considered the most suitable for our design (left gel image, lane 1). We next modified OMe-C10 in the catalytic core (M34-2) and altered the design of the binding arm (LNA and 2′-OMe modified, termed M34-1) to avoid RNase H recognition. Both designs worked well for the *MALAT-1* partial sequence, M-Rs-21nt (right gel image, lanes 2 and 3). The negative controls (M34-1_inv and M34-1_mut) did not exhibit cleavage activity. c) Cleavage assay using X8–17 against the longer substrate (70 nt, termed M-Rs-70nt). A site-specific cleavage product (34 nt) was clearly observed under both multiple-turnover (m) and single-turnover (s) conditions. The cleavage assay was conducted under the same conditions as those mentioned in Supplementary Fig. 3 but with a different X8–17 concentration under single-turnover (0.2 µM X8–17 was incubated with 0.1 µM substrate).

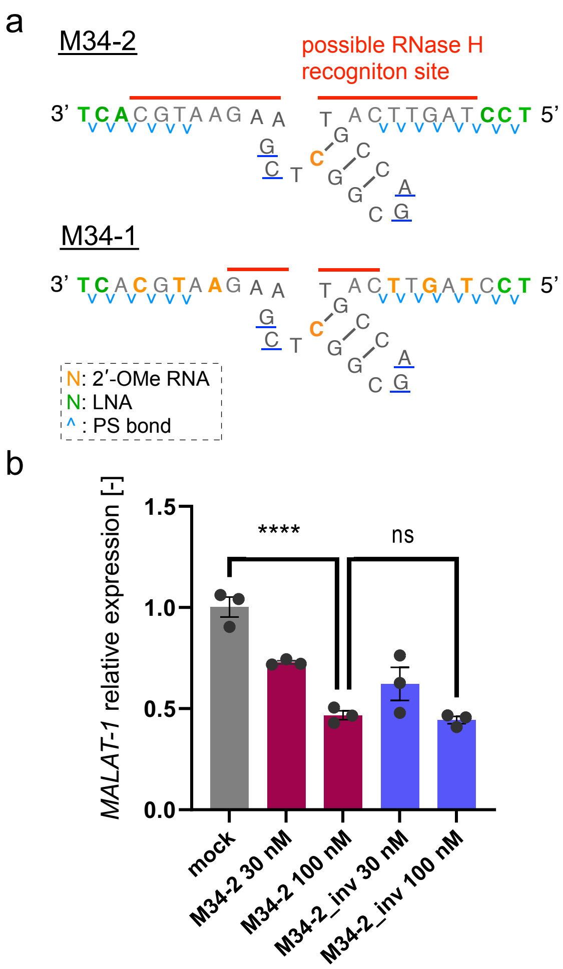

Supplementary Fig. 7. *MALAT-1* knockdown assay of X8–17.

a) The X8–17 binding arm was designed similarly to the gapmer structure of ASOs (M34-2). As a reference, M34-1 (used in Fig. 4) is shown. M34-2 was long enough to recruit RNase H (red bar). b) The result of the knockdown assay using Hepa1c1c cells. M34-2 and its negative control (M34-2_inv) exhibited comparable knockdown activity, indicating that mainly RNase H-mediated knockdown (antisense effect) occurred. The results of the experiment were analyzed using one-way ANOVA and Dunnett′s test (ns, not significant, **** $P < 0.0001$).

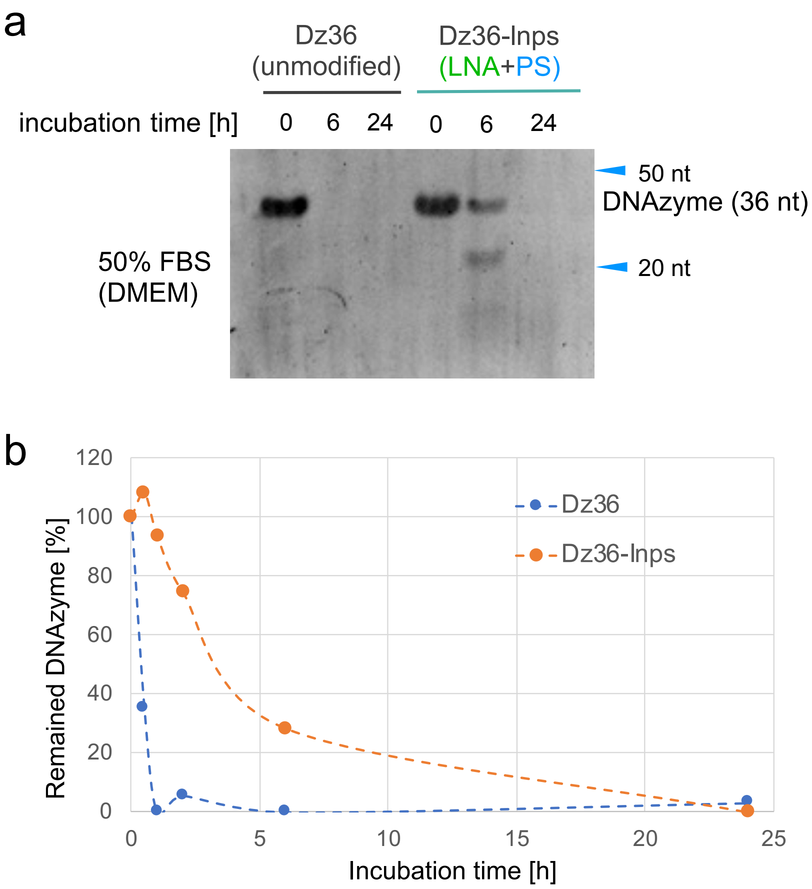

Supplementary Fig. 8. Expansion of biostability in fetal bovine serum (FBS).

a) Biostability assays for unmodified 8–17 DNAzyme (Dz36) and 8–17 DNAzyme with LNA and PS modifications (Dz36-lnps). The incubation time was extended to 6 h or 24 h in 50% FBS and DMEM solution at 37 °C. The samples were analyzed using urea-PAGE followed by SYBR Gold staining. b) Time-dependent biostability in 50% PBS, showing relative levels of remaining Dz36 and Dz36-lnps. The results are based on data shown in Fig. 2e and Supplementary Fig. 6a. Dz36 had decomposed completely within 1 h, whereas 30% of the Dz36-lnps was remaining after 6 h.

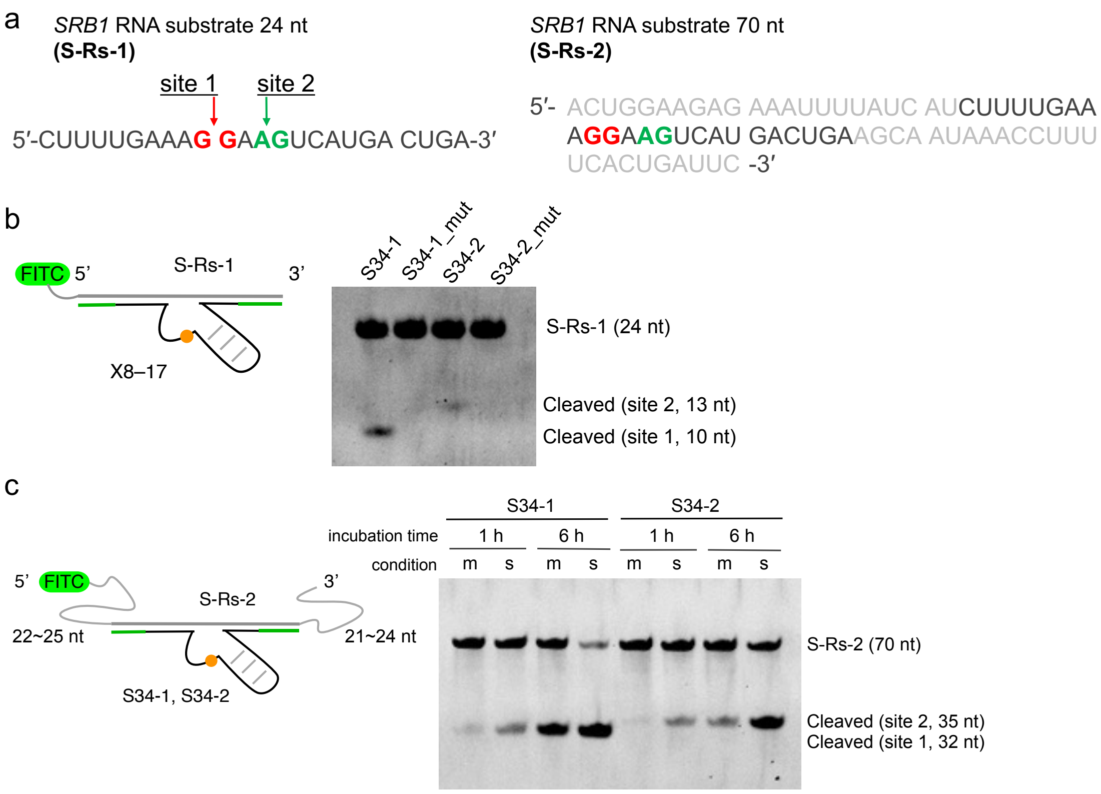

Supplementary Fig. 9. *SRB1* cleavage assay analyzed using urea-PAGE.

a) *SRB1* partial sequences used for the assay. RNA substrates of 24 nt and 70 nt were prepared to confirm the site-specific cleavage and the effects of the more structured substrate (secondary structure). FITC was conjugated to its 5′ end. Cleavage sites are shown in red and green. b) X8–17 cleavage assay. S34-1 and S34-2 cleaved the substrate, indicated by the cleaved products (10 and 13 nt), as expected. For the negative controls (S34-1_mut and S34-2_mut), cleaved products were not observed. c) Cleavage assay of X8–17 against the longer substrate (70 nt). Site-specific cleavage products (32 nt and 35 nt) were clearly observed under both multiple-turnover (m) and single-turnover (s) conditions. The cleavage assay was conducted under the same conditions as in Supplementary Fig. 3 but with a different X8–17 concentration in the single-turnover assay (0.2 μM X8–17 was incubated with 0.1 μM substrate).

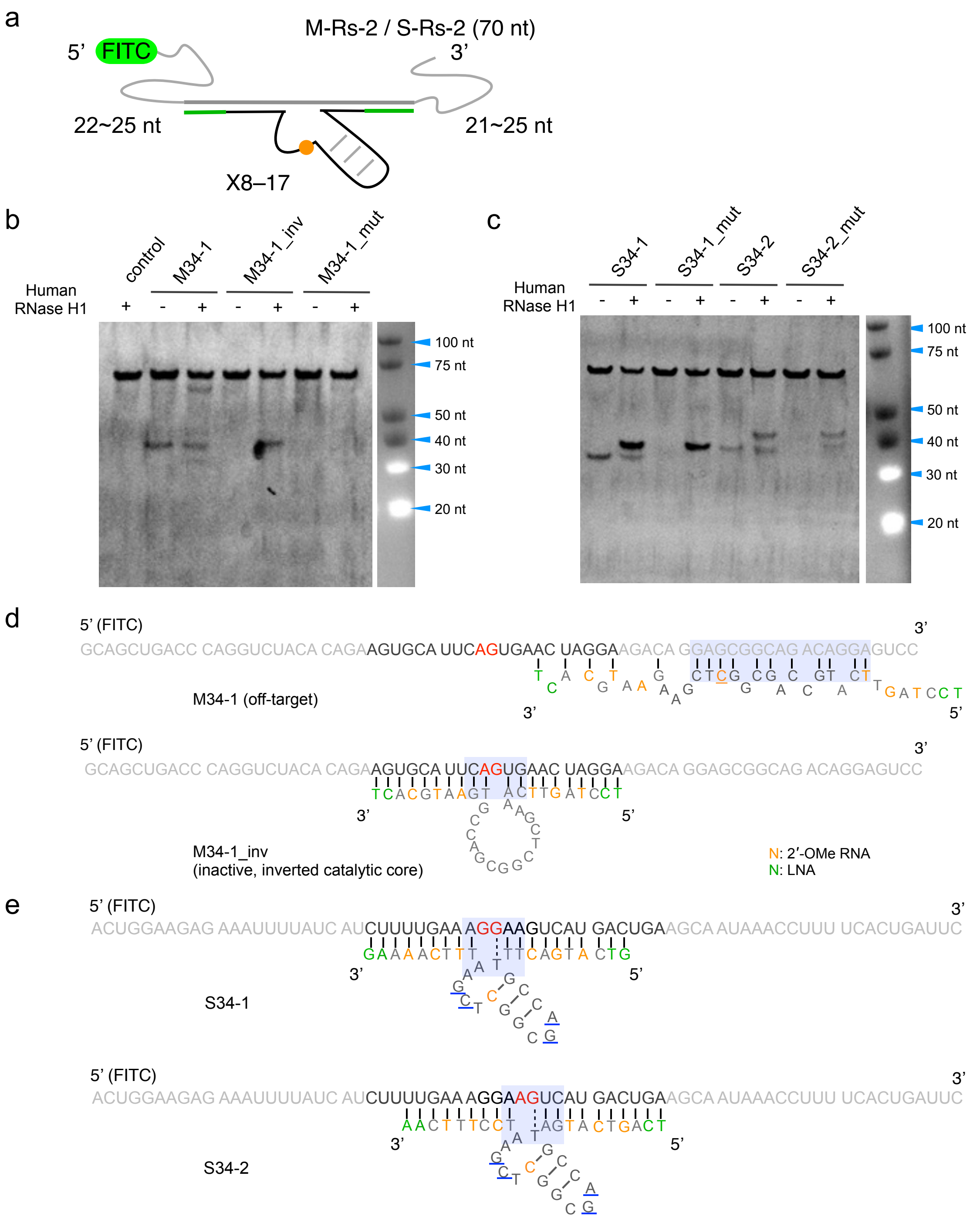

Supplementary Fig. 10. RNase H-mediated cleavage of *MALAT-1* and *SRB1* in the presence of X8–17s and their negative controls.

a) For the RNase H assay, X8–17s and long substrates (Supplementary Figs. 4a and 7a) were co-annealed. b) Human RNase H1 assay with *MALAT-1* targeting X8–17 and its substrate. DNAzyme mediated cleavage of M34-1 was observed whether RNase H was present (lane 3) or absent (lane 2). b, d) RNase H-mediated cleavage bonds were observed for ca. 50–70 nt (lanes 3, 7) and ca. 40 nt (lane

5) substrate sequences. We hypothesize that M34-1 and M34-1_mut were bound to the different site of the substrate via their catalytic-core sequence (upper figure of d); off-target effect) and that M34-1_inv was canonically bound to the cleavage site (lower figure of d); via A-T base-pairing caused by sequence inversion). Blue filled squares: potential RNase H recognition sites. c) Human RNase H1 assay with *SRB1* targeting X8–17 and its substrate. DNAzyme-mediated cleavage of S34-1 and S34-2 was observed whether RNase H was present (lanes 2 and 6) or absent (lanes 1 and 5). c, e) RNase H-mediated cleavage bonds were observed for substrate sequence lengths of < 40 nt (lanes 2 and 4) and < 50 nt (lane 6, 8). It is hypothesized that S34-1 and S34-2, and their negative controls, were recognized by RNase H at DNAzyme-mediated cleavage sites with G–T wobble base-pairing (blue square). Importantly, the difference in RNase-mediated bond cleavage between X8–17s and their negative controls was negligible.

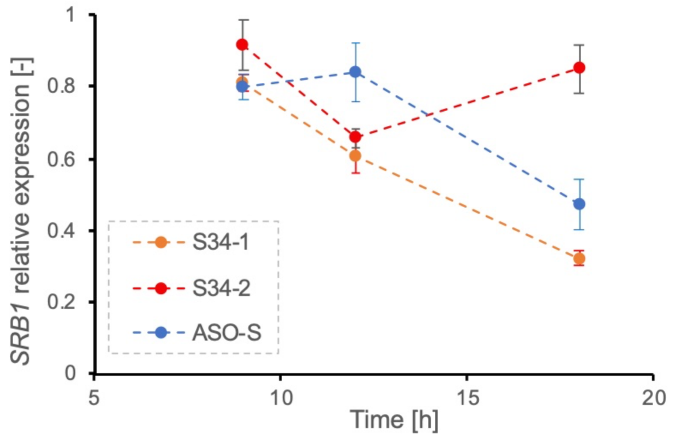

Supplementary Fig. 11. Time-dependent knockdown of *SRB1* RNA in Hepa1c1c7 cells.
The relative expression of *SRB1* was calculated via the ΔΔCt method with *GAPDH* as a housekeeping gene. The figures show changes in knockdown activity of X8–17s for *SRB1* (S34-1 and S34-2, at 100 nM each) and ASO targeting *SRB1* (ASO-S, at 10 nM) with the incubation time after transfection.

Uncropped Full Gel Images

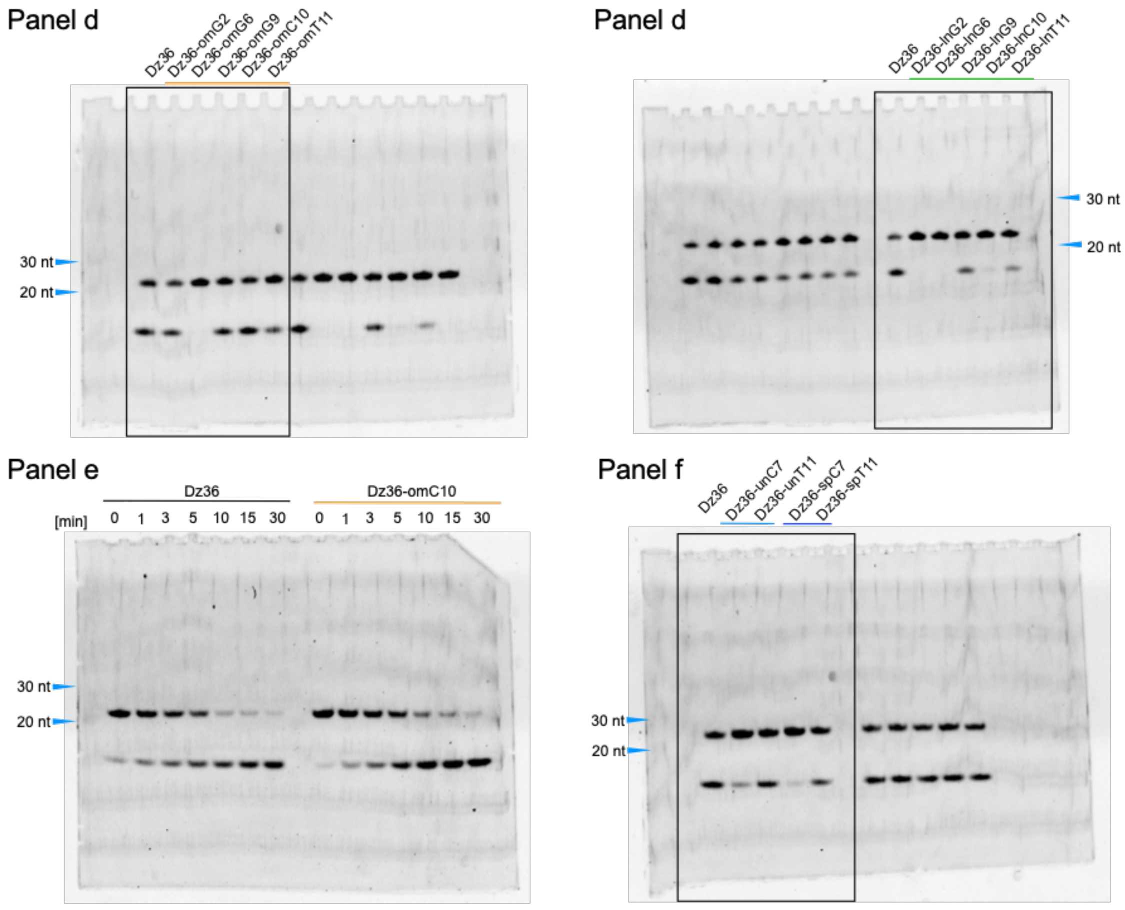

Uncropped full gel image of Fig. 1. Black frames indicate the lanes used for figures.

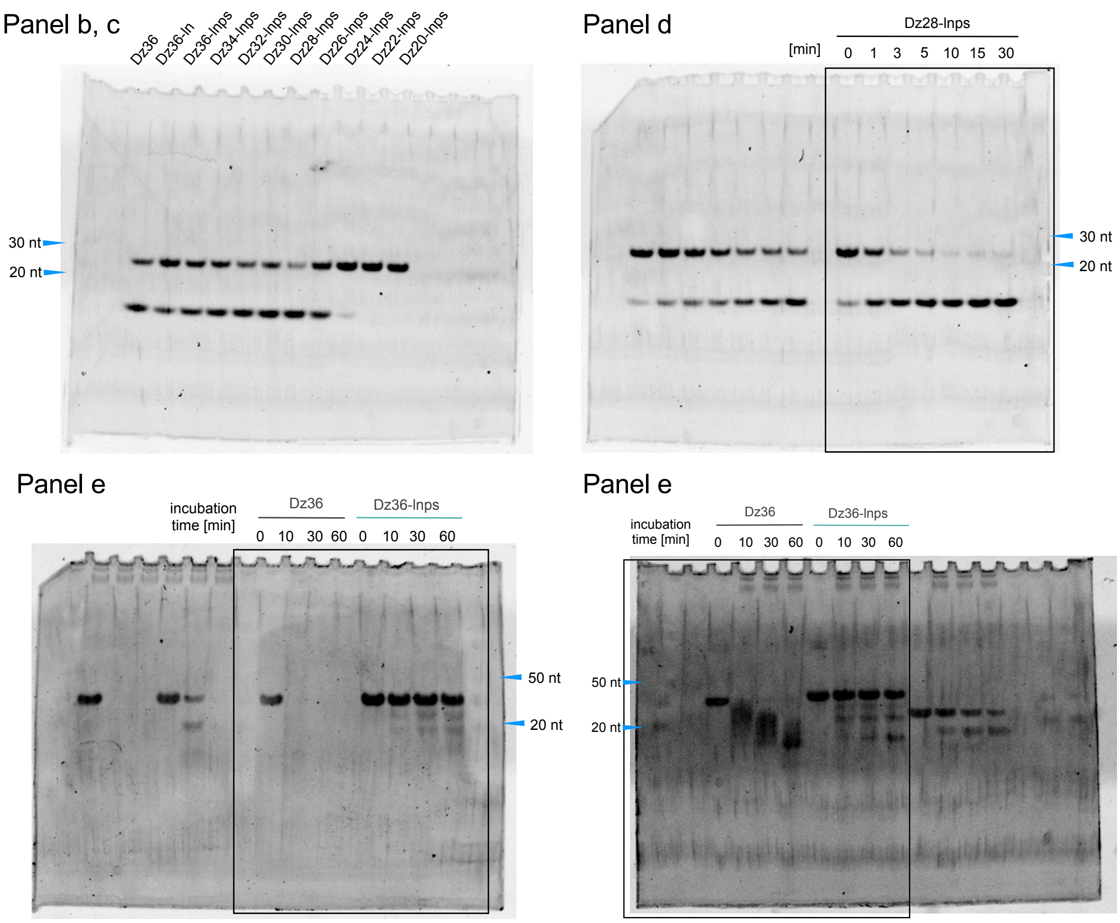

Uncropped full gel image of Fig. 2. Black frames indicate the lanes used for figures.

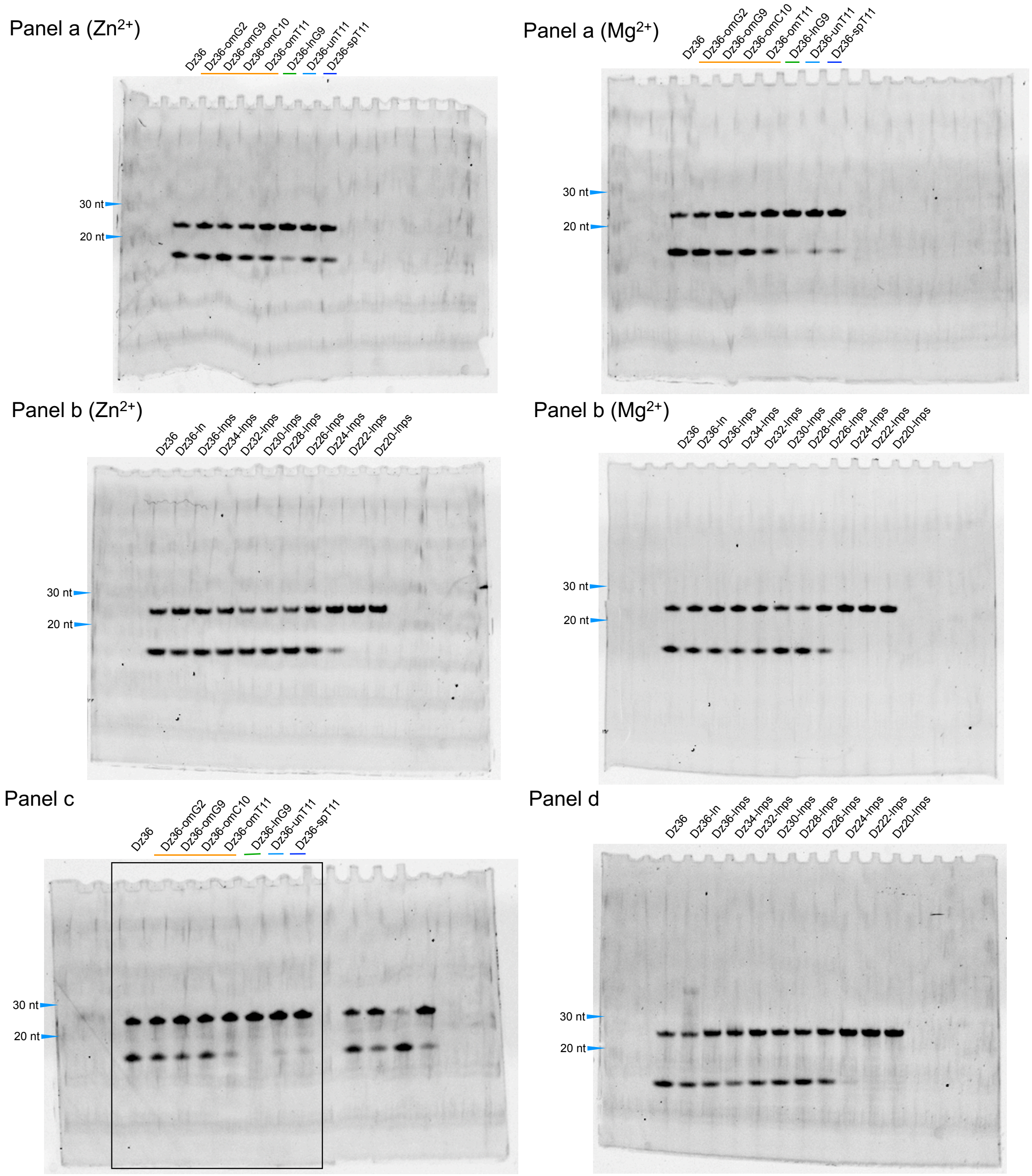

Uncropped full gel image of Fig. 3. Black frames indicate the lanes used for figures.

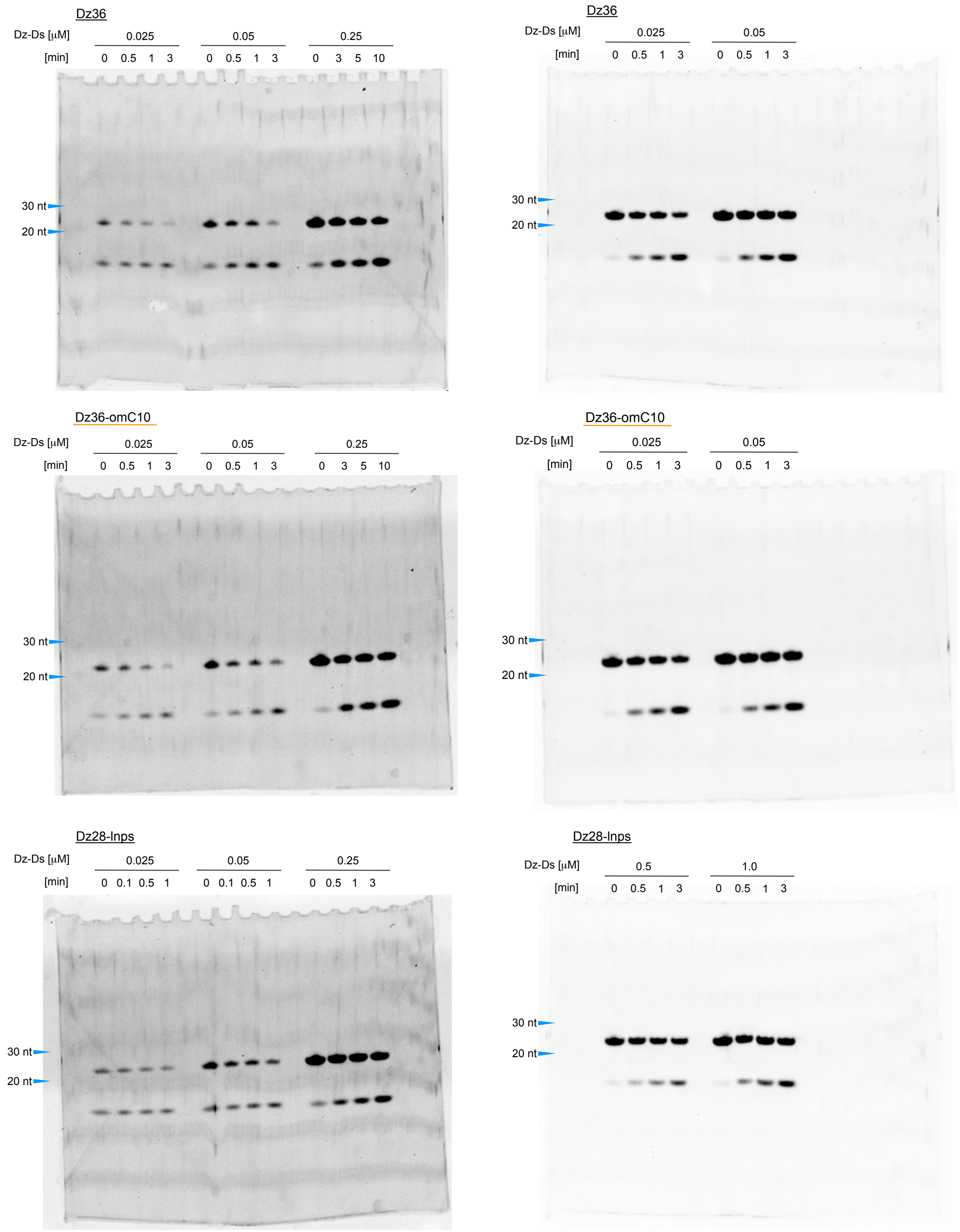

Uncropped full gel image of Fig. S1.

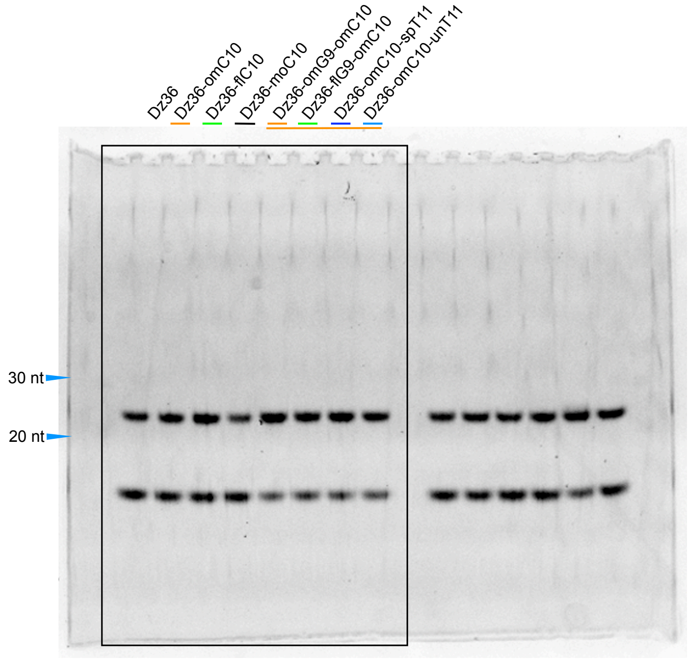

Uncropped full gel image of Fig. S2. Black frames indicate the lanes used for figures.

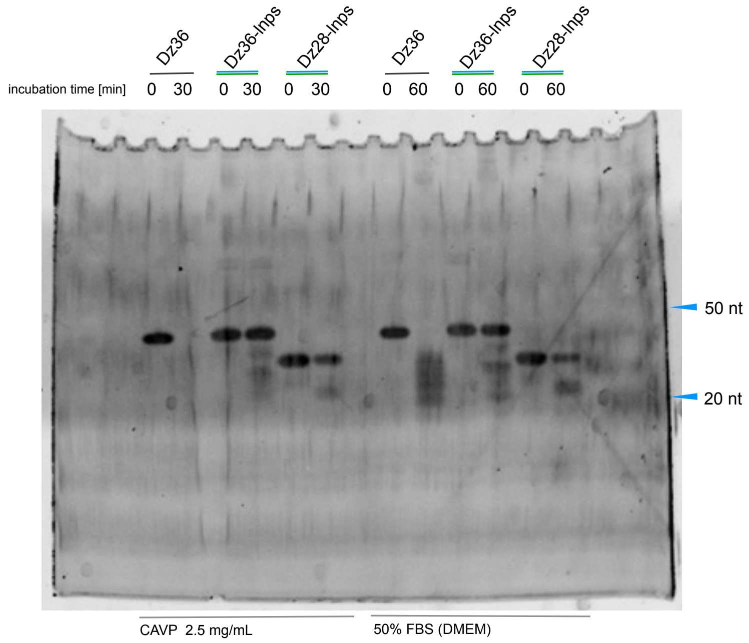

Uncropped full gel image of Fig. S3.

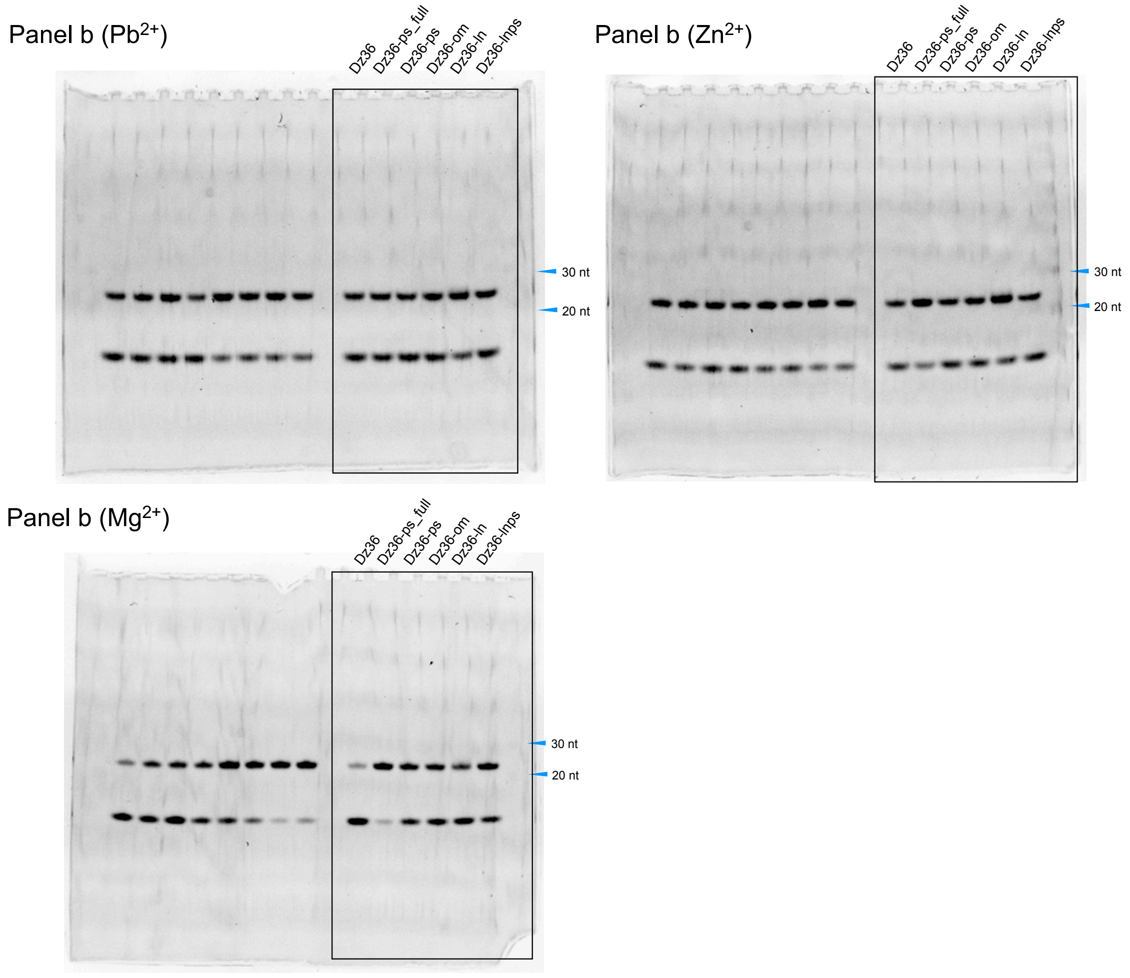

Uncropped full gel image of Fig. S2. Black frames indicate the lanes used for figures.

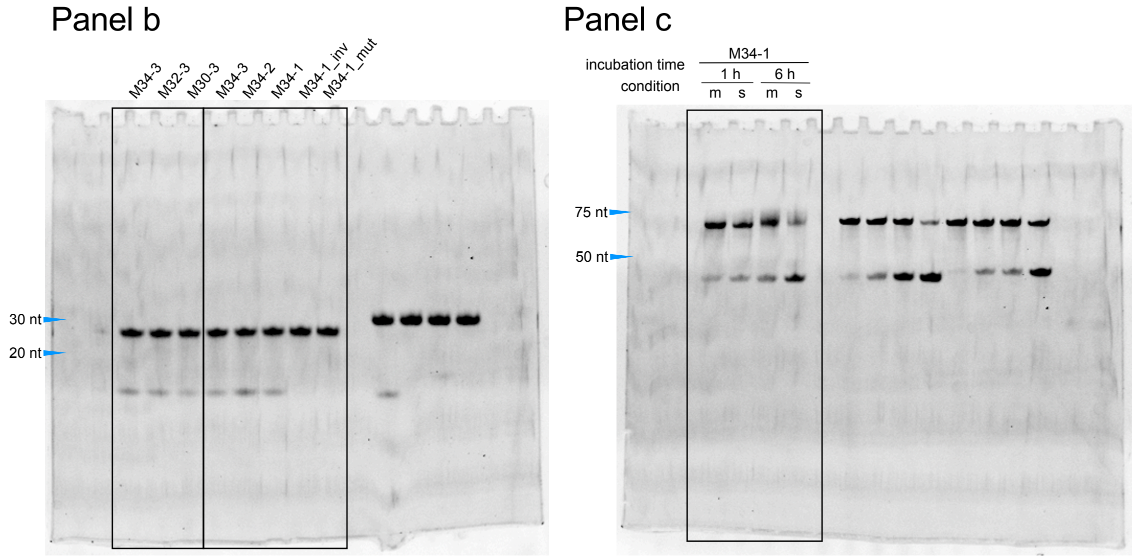

Uncropped full gel image of Fig. S4. Black frames indicate the lanes used for figures.

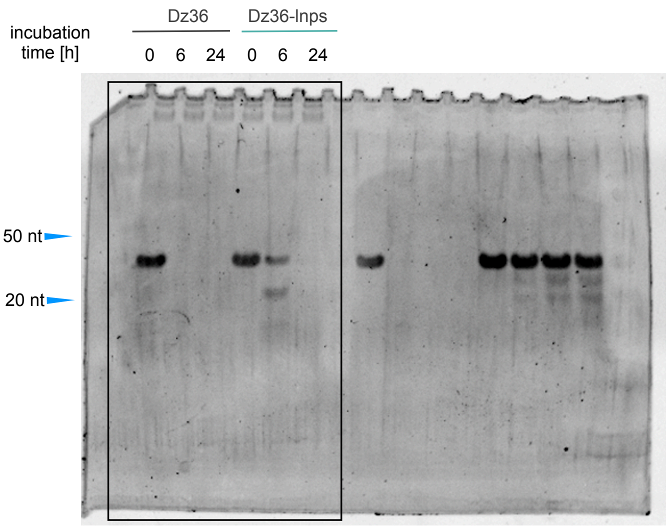

Uncropped full gel image of Fig. S6. Black frames indicate the lanes used for figures.

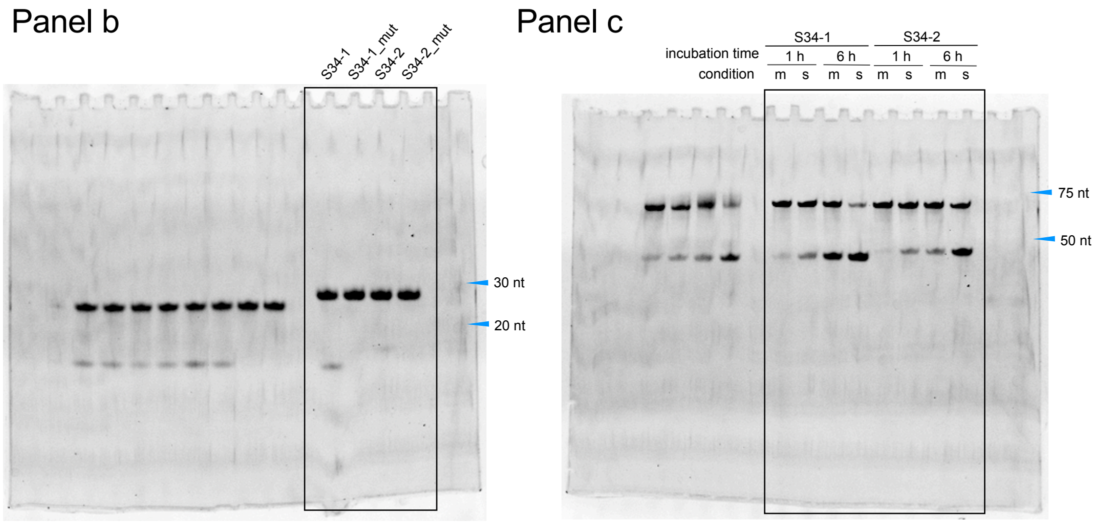

Uncropped full gel image of Fig. S7. Black frames indicate the lanes used for figures.

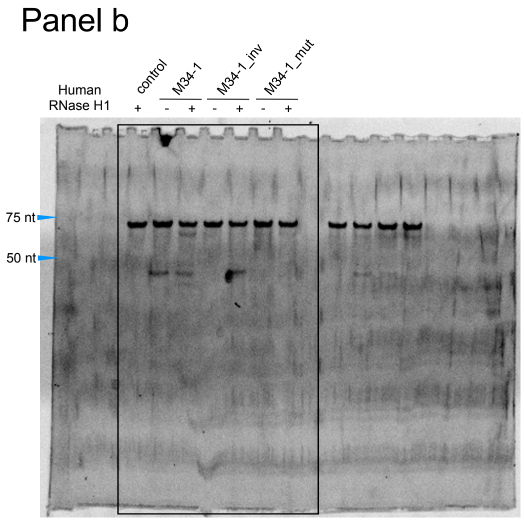

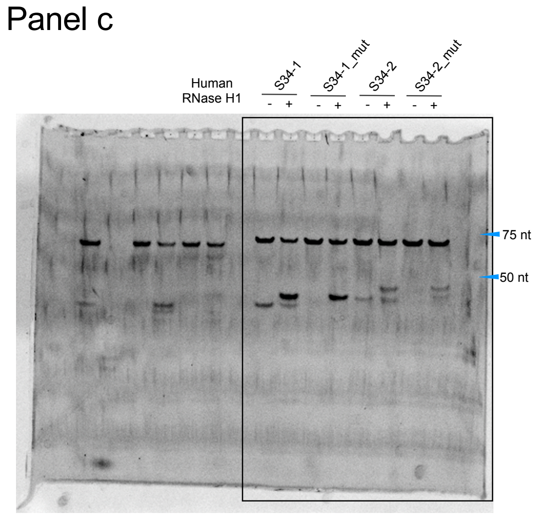

Uncropped full gel image of Fig. S8. Black frames indicate the lanes used for figures.

Supplementary References

1. Liu, H. et al. Crystal structure of an RNA-cleaving DNAzyme. *Nat. Commun.* **8**, 2006 (2017).
2. Adamiak, D. A., Rypniewski, W., Mileckl, R., J., Adamiak, R. W. The 1.19 Å X-ray structure of 2′-O-Me(CGCGCG)$_2$ duplex shows dehydrated RNA with 2-methyl-2,4-pentanediol in the minor groove. *Nucleic Acids Res.* **29**, 4144–4153 (2001).
3. Petersen, M., Bondensgaard, K., Wengel, J., Jacobsen J. P. Locked nucleic acid (LNA) recognition of RNA: NMR solution structures of LNA:RNA hybrids. *J. Am. Chem. Soc.* **124**, 5974–5982 (2002).
